# Morphological profiling of tubercle bacilli identifies drug pathways of action

**DOI:** 10.1073/pnas.2002738117

**Published:** 2020-07-17

**Authors:** Trever C. Smith, Krista M. Pullen, Michaela C. Olson, Morgan E. McNellis, Ian Richardson, Sophia Hu, Jonah Larkins-Ford, Xin Wang, Joel S. Freundlich, D. Michael Ando, Bree B. Aldridge

**Affiliations:** ^a^Department of Molecular Biology and Microbiology, Tufts University School of Medicine, Boston, MA 02111;; ^b^Center for Integrated Management of Antimicrobial Resistance (CIMAR), Tufts University, Boston, MA 02111;; ^c^Department of Biological Engineering, Massachusetts Institute of Technology, Cambridge, MA 02139;; ^d^Roxbury Latin School, West Roxbury, MA 02132;; ^e^Department of Bioinformatics and Computational Biology, University of Maryland, Baltimore County, Baltimore, MD 21250;; ^f^Tufts University School of Graduate Biomedical Sciences, Boston, MA 02111;; ^g^Laboratory of Systems Pharmacology, Harvard Medical School, Boston, MA 02115;; ^h^Department of Pharmacology, Physiology, and Neuroscience, Rutgers University–New Jersey Medical School, Newark, NJ 07103;; ^i^Division of Infectious Disease, Department of Medicine, Rutgers University–New Jersey Medical School, Newark, NJ 07103;; ^j^Ruy V. Lourenco Center for the Study of Emerging and Re-emerging Pathogens, Rutgers University–New Jersey Medical School, Newark, NJ 07103;; ^k^Applied Science Team, Google Research, Mountain View, CA 94043;; ^l^Department of Biomedical Engineering, Tufts University School of Engineering, Medford, MA 02155

**Keywords:** tuberculosis, drug discovery, cell morphology, high throughput

## Abstract

Morphological profiling is a method to classify target pathways of antibacterials based on how bacteria respond to treatment through changes to cellular shape and spatial organization. Here we utilized the cell-to-cell variation in morphological features of *Mycobacterium tuberculosis* bacilli to develop a rapid profiling platform called Morphological Evaluation and Understanding of Stress (MorphEUS). MorphEUS classified 94% of tested drugs correctly into broad categories according to modes of action previously identified in the literature. In the other 6%, MorphEUS pointed to key off-target activities. We observed cell wall damage induced by bedaquiline and moxifloxacin through secondary effects downstream from their main target pathways. We implemented MorphEUS to correctly classify three compounds in a blinded study and identified an off-target effect for one compound that was not readily apparent in previous studies. We anticipate that the ability of MorphEUS to rapidly identify pathways of drug action and the proximal cause of cellular damage in tubercle bacilli will make it applicable to other pathogens and cell types where morphological responses are subtle and heterogeneous.

## Significance

Tuberculosis is a leading cause of death in the world and requires treatment with an arduous multidrug regimen. Many new tuberculosis drugs are in development, and the drug development pipeline would benefit from more rapid methods to learn drug mechanism of action and off-target effects. Here we describe a high-throughput imaging method for rapidly classifying drugs into categories based on the primary and secondary mechanisms of cellular damage caused by different antibacterials called Morphological Evaluation and Understanding of Stress (MorphEUS). We anticipate that MorphEUS will assist in rapidly pinpointing pathway of action of antibacterials for tuberculosis and other bacterial infections.

*Mycobacterium tuberculosis* (Mtb), the causative agent of tuberculosis (TB), remains a global menace, killing ∼4,000 people a day (1). Tuberculosis treatment is lengthy, lasting from 4 mo to over a year (1). The difficult regimen, rate of relapse, and incidence of drug resistant Mtb has motivated a significant effort to develop new antibacterial compounds that are effective in sterilizing Mtb infection (2). Many new drug classes and derivative compounds have been developed (2), but rapidly identifying the primary and secondary pathways of action is often a protracted process due to the difficulty in generating resistant mutants and dissecting the broad-reaching metabolic effects of drug treatment (3). Furthermore, bacterial cells can elicit dynamic responses in multiple pathways both on and off target, some of which are specific to bacterial growth environment and treatment dose, thereby confounding mechanism of action studies (3–6). A rapid method to interrogate the pathways of drug action in Mtb could be used to increase throughput and complement traditional molecular, genetic, and metabolic approaches to shorten TB’s drug development timeline.

In other bacterial species such as *Escherichia coli*, *Bacillus subtilis*, and *Acinetobacter baumannii*, profiling of cytological changes in response to drug treatment has yielded a rapid and resource-sparing procedure to determine drug mechanism (7–9). This method, known as bacterial cytological profiling (BCP), is based on the principle that bacteria respond to drug treatment with morphological changes that are characteristic of the drug’s pathway of action. BCP groups drugs with similar mechanisms of action by clustering profiles of drug-treated bacteria using multivariate analyses including principal component analysis (PCA) (7–9). BCP is efficient and rapid because cytological features can be derived from high-throughput images of stained, fixed samples.

To accelerate the drug development pipeline for tuberculosis, we aimed to develop a method to understand drug pathways of action in Mtb using rapid and cost-effective tools. We hypothesized that BCP could be utilized to map pathways of drug action in Mtb. We found that the morphological shifts in drug-treated Mtb were subtle and exhibited cell-to-cell variation that obscured the ability of traditional BCP pipelines to classify drug profiles. To utilize cellular heterogeneity as a distinguishing feature of drug response and to overcome Mtb’s morphological subtleties, we developed an imaging and analysis pipeline. Here we describe this tool, called Morphological Evaluation and Understanding of Stress (MorphEUS).

Using MorphEUS in Mtb, we were able to classify the cellular targets of 34 known antibacterials and three noncommercial compounds in a blinded study. Antibacterial compounds may impact multiple pathways either through off-target or secondary effects. In some cases, these effects are thought to be major contributors to bactericidal activity (10–13). These polypharmacologies can be readily observed using MorphEUS because it captures how cells physically deteriorate as different cellular pathways are inhibited. Using MorphEUS, we identified secondary effects for two clinically relevant TB drugs, moxifloxacin and bedaquiline. We propose that MorphEUS will be useful in classifying drug action for new compounds in Mtb and in other pathogens where morphological responses are subtle and heterogeneous.

## Results

### Antibacterial Treatment Induces Drug-Specific Morphological Response in Mycobacteria.

**Fig. 1.**
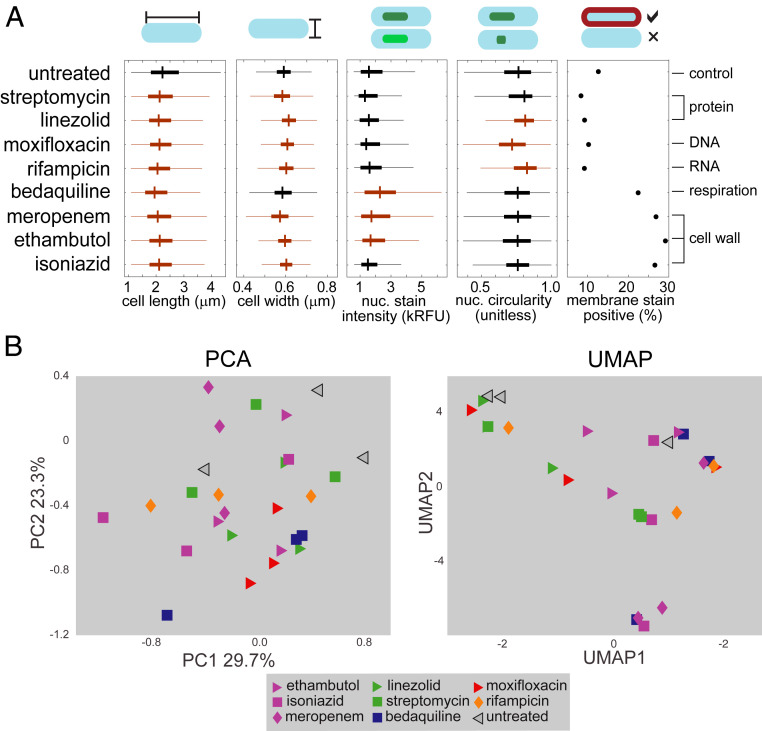
Drug treatment induces subtle morphological changes in Mtb. (*A*) A comparison of select Mtb morphological features across eight antibiotic treatments and untreated control (*n* = 1,625 to 3,983). The boxes mark the 25th to 75th percentiles, and the whiskers extend the range of parameters that are not outliers. Orange boxes indicate *P* < 0.05 compared to untreated control (at the top), whereas black boxes are not significantly different from untreated using a Kruskal–Wallis test. (*B*) PCA (*Left*), and UMAP (*Right*) of eight drug treatments at high dose (3× IC90) resulting from established analysis methods (7–9, 47) using feature medians. The treatment nodes are color coded based on the known broad cellular target as determined by literature review (*SI Appendix*, Table S1).

We hypothesized BCP could be used to classify drug pathways in Mtb as it does for other bacterial species (7–9). Guided by these cytological profiling methods (7–9), we treated Mtb grown in standard rich growth medium with a high drug dose (3× the 90% inhibitory concentration [IC90]) for 17 h (∼1 doubling time). We imaged fixed, membrane-stained (FM4-64FX), and nucleoid-stained (SYTO 24) Mtb in biological triplicate to generate a dataset of morphological features from Mtb treated with 34 antibacterials that encompass a wide range of drug classes according to published findings (*SI Appendix*, Table S1). Using image segmentation and analysis, we quantified 25 morphological features per treatment group (*SI Appendix*, Table S2). We observed significant differences among treatment groups in features such as cell shape, nucleoid shape, and staining intensity (Fig. 1*A*). However, the resulting morphological profiles from drug treatment did not cluster based on broad drug target categories using either PCA or uniform manifold approximation and projection (UMAP) (Fig. 1 *B*, *Left* and *Right*, respectively; *SI Appendix*, Figs. S2*A* and S3). We conclude that unlike *Mycobacterium smegmatis* (Movies S1–S3), *E. coli*, and *B. subtilis* (8, 9), Mtb does not exhibit striking physical differences that readily distinguish drugs targeting dissimilar cellular pathways (Fig. 1).

One explanation for the poor performance of BCP in Mtb may be the significant cell-to-cell variation in morphological features (Fig. 1 and *SI Appendix*, Fig. S1). This inherent heterogeneity is consistent with the variable nature of Mtb, which on the single-cell level exhibits heterogeneity through asymmetric growth and division, differential drug susceptibility, and metabolic state (14–18). Cell-to-cell variation is most apparent in the ability of Mtb bacilli to take up stains. For example, only ∼10% of untreated bacilli are stain positive, whereas the proportion increases to ∼30% when treated with cell wall-acting antibacterials (Fig. 1*A*). We speculated that variation itself was an important feature of drug response that should be captured in the profiling of drug mechanism of action.

### Morphological Profiling of Mtb Is Improved by Explicit Incorporation of Parameters of Cellular Variation.

**Fig. 2.**
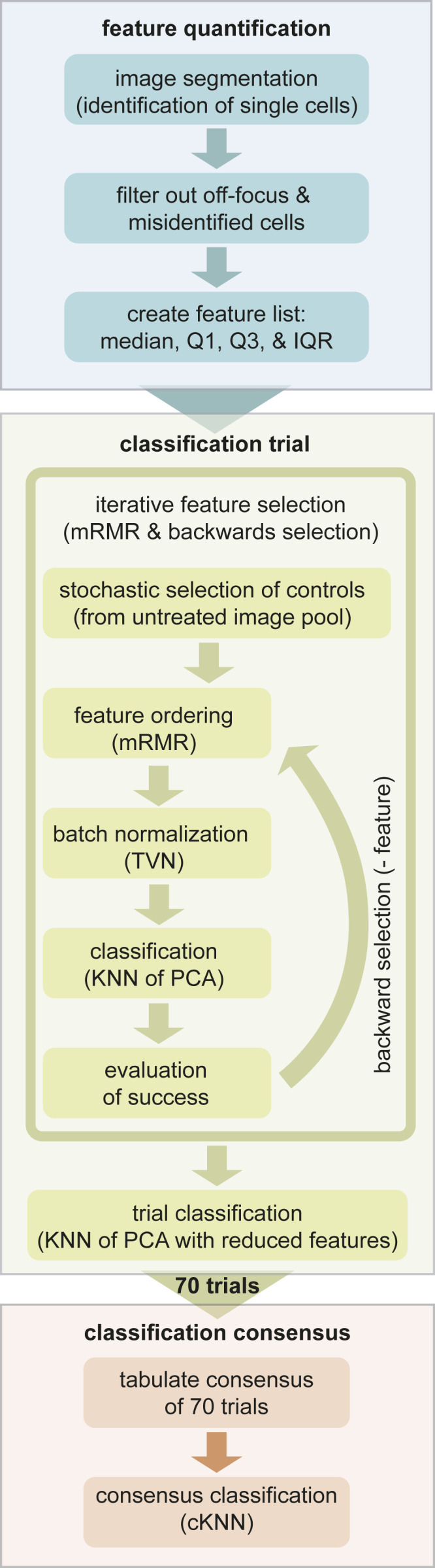
Computational pipeline for MorphEUS. The MorphEUS pipeline is composed of three steps: feature quantification (blue), classification trials (green), and classification consensus (orange). The main components of each step are highlighted as boxes within each of the three groups. A detailed description of each step is described in *Materials and Methods*.

**Fig. 3.**
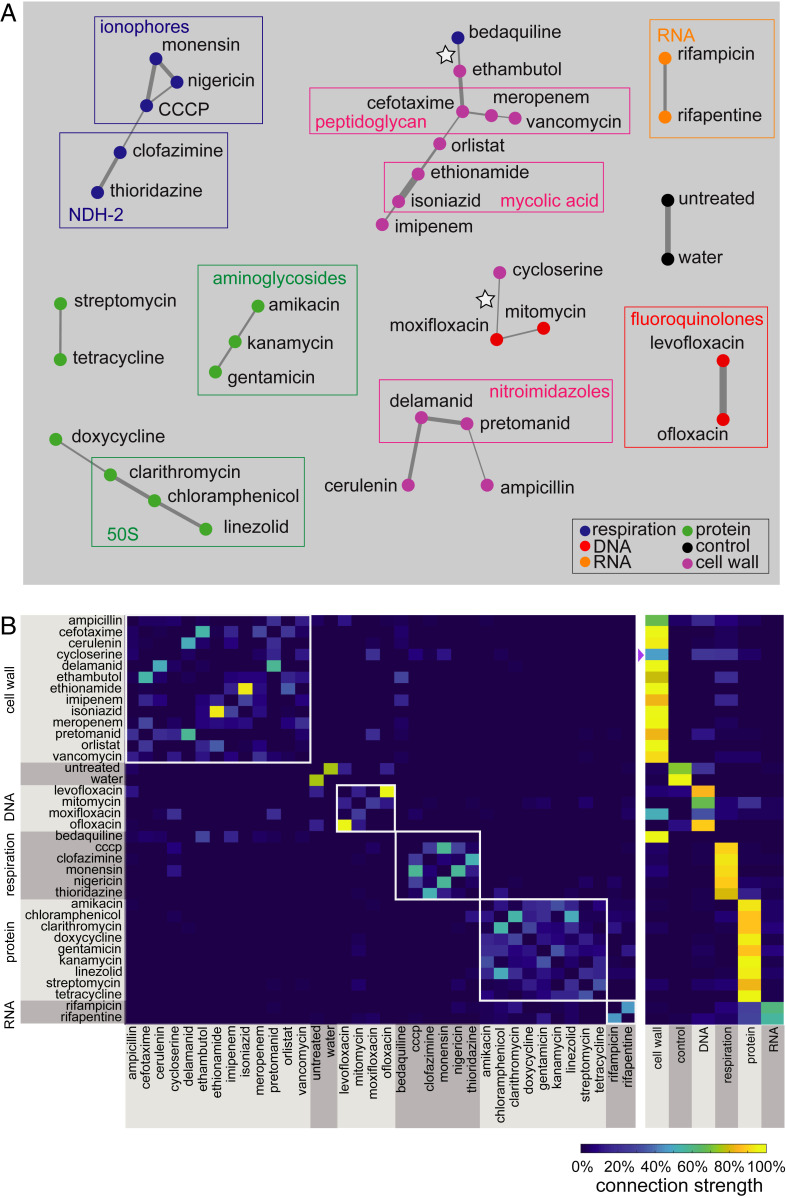
MorphEUS classifies antibacterial compounds by pathway of action. (*A*) cKNN map of the joint dose profile displaying connections that occur in at least 17% of the classification trials. Drugs within each broad category are represented by nodes of the same color, illustrating whether morphological profiles were similar among drugs acting on the same pathway. Edge thickness indicates the connection frequency for a given connection between two treatment profiles. Rectangles drawn around groups of drugs indicate clustering of drugs that share similar targets within the designated broad category. White stars mark unexpected connections between antibacterials belonging to two different broad categories. (*B*) cKNN matrix of drug nearest neighbor pairings corresponding to *A* by specific drugs (*Left*) and broad categorization (*Right*). The broad drug target categorizations are indicated to the left of the drug names and on the bottom axis of the heat map on the right. A purple triangle is placed next to the broad categorization for the weakly categorized cell wall acting drug cycloserine.

To capture cell-to-cell variation, we developed an analysis pipeline for Mtb that incorporates variation as an important class of features to discriminate drug target pathways (*SI Appendix*, Table S1 and S2 and Figs. S2, S4, and S5; Fig. 2; and ref. 19). This analysis formulation also addresses the subtlety of cytological changes by taking into account the full dimensionality of the data to produce discrete classifications. The exploitation of feature variation provided increased resolution to distinguish drug categories. For example, when treated with isoniazid, Mtb nucleoid stain intensity was less variable than when treated with bedaquiline or meropenem (Fig. 1*A*). We accounted for a fragile feature selection process (in which several sets of features may achieve similar model accuracy) by performing a series of classification trials (Fig. 2). The resulting analysis was visualized using a network web or matrix describing the frequency of drug–drug links (Fig. 3). The network (connectivity) webs depict the strongest treatment (drug–drug) connections, whereas the matrix displays all of the pairwise drug similarities. Relationships between treatments on the network maps may appear to have lower connection strengths since we only display treatment pairs that most frequently profile together. In other words, a treatment that produces a morphological profile similar to three other drugs in the same broad category will have weaker individual connections compared to a drug that results in a response similar to just one other treatment (Fig. 3*B*). We measured morphological responses in bacilli treated with both high and low doses of drugs (*SI Appendix*, Fig. S5). Some drugs elicited stronger cytological shifts at inhibitory doses rather than subinhibitory doses, while other treatments resulted in the opposite behavior (such as ethionamide at high dose and rifampicin at low dose) (*SI Appendix*, Fig. S5). This observation led us to hypothesize that combining the feature sets from both the high- and low-dose profiles (into a joint dose profile) would generate a more descriptive profile for classification of each drug. The cross-validation rate for the joint dose analysis was improved (76%) compared to low- and high-dose analysis (62 and 68%, respectively; Fig. 3 and *SI Appendix*, Fig. S5). We therefore incorporated joint (high- and low-dose) drug profiles as a default in our analysis pipeline. Together, we refer to this analysis pipeline (Fig. 2) as MorphEUS.

Using MorphEUS, drugs in the same broad categories are generally grouped together (94% accurate categorization; Fig. 3). Furthermore, within the broad categories, drugs sharing target pathways were found to have stronger connections to one another. For example, within the cell wall-acting category, strong connections were observed between ethionamide and isoniazid (inhibitors of the enzyme InhA); delamanid and pretomanid (nitroimidazole drug class, which inhibits the synthesis of mycolic acids); and meropenem, cefotaxime, and vancomycin (all peptidoglycan inhibitors) (Fig. 3 and *SI Appendix*, Table S1). We also observed strong connectivity between inhibitors of cellular respiration with the ionophores CCCP, monensin, and nigericin forming stronger connections with each other compared to clofazimine and thioridazine [both shown to target NDH-2 of the electron transport chain (20, 21)]. Strong connections among protein synthesis inhibitors that target the 50S ribosomal subunit were also observed among clarithromycin, chloramphenicol, and linezolid (*SI Appendix*, Table S1). Finally, the fluoroquinolones levofloxacin and ofloxacin grouped together as did rifampicin and rifapentine (inhibitors of transcription). Ampicillin’s strongest connection was with cell wall-acting pretomanid and not peptidoglycan-targeting β-lactams. This may be due to the expression of β-lactamases by Mtb which inactivates β-lactams like ampicillin (22, 23), coupled with the substantially greater in vitro catalytic efficiency of ampicillin as a BlaC substrate as compared to the other two β-lactams (24), thereby diminishing its profiling with other peptidoglycan inhibiting drugs.

### Morphological Response to Treatment Reflects Key Off-Target Effects.

Among the 34 antibacterials profiled, only cycloserine and bedaquiline were miscategorized by general drug group (white stars in Fig. 3*A*); e.g., their profiles most strongly linked to an antibacterial from a different broad drug category. Cycloserine is a cell wall-acting drug that inhibits the formation of peptidoglycan (25). Cycloserine weakly profiled with the category of cell wall-acting antibacterials, but its strongest connection to an individual antibacterial was with the fluoroquinolone moxifloxacin (DNA-damaging; white star in Fig. 3). Given that both mitomycin and moxifloxacin are DNA-acting antibacterials (*SI Appendix*, Table S1), we hypothesized that the connection between cycloserine and moxifloxacin was mediated by an off-target DNA damaging effect from cycloserine treatment. To test this hypothesis, using data from previous studies (26, 27) we compared the transcriptional profiles of cells treated with cycloserine to the transcriptional profiles of Mtb treated with 16 compounds (including cycloserine and moxifloxacin) targeting different pathways in Mtb (*SI Appendix*, Fig. S6). Comparisons between expression profiles were performed using hierarchical clustering with Pearson correlation as the distance metric (28, 29) (*Materials and Methods*). The Pearson distance is calculated using covariances between samples and is equivalent to cosine correlation for high dimensional variables, such as expression levels of multiple genes after drug treatment. Because Pearson distances cluster based on the patterns of expression rather than absolute expression levels, it is an appropriate clustering metric to assess trends in gene expression among samples from different studies (28, 29). We focused our analysis on genes involved in the SOS response of mycobacteria because the SOS response is up-regulated when DNA damage occurs in bacteria (30–32). We observed that cycloserine, unlike known DNA targeting agents (mitomycin, levofloxacin, ofloxacin, and moxifloxacin), does not induce an SOS response suggesting that cycloserine treatment does not cause DNA damage in Mtb (*SI Appendix*, Fig. S7).

An alternative hypothesis is that the cycloserine-to-moxifloxacin connection is driven by unexpected off-target effects of moxifloxacin, such as cell wall damage. Moxifloxacin’s morphological profiles are highly dose dependent, resembling the other fluoroquinolones at low dose but not at high dose or in joint dose profiles (*SI Appendix*, Fig. S5 and Fig. 3). This shift away from other fluoroquinolones by moxifloxacin suggests that there is an off-target or secondary effect at high dose. We evaluated the transcriptional response of Mtb treated with moxifloxacin with respect to cell wall damage by focusing on the *iniBAC* operon (*SI Appendix*, Fig. S6*A*). The *iniBAC* operon was first investigated because it is known to be up-regulated upon chemical inhibition of cell wall synthesis and is therefore used to screen for cell wall-acting compounds (33, 34). As expected, we observed an induction in *iniB*, the first gene in the *iniBAC* operon, in Mtb cells treated with cycloserine. Similarly, we found that moxifloxacin-treated Mtb demonstrated a mild but significant increase in *iniB* expression (*SI Appendix*, Fig. S6*B*). Further analysis of the transcriptional response of 41 genes involved in cell wall damage and peptidoglycan biosynthesis (33, 35, 36) revealed that the profiles of moxifloxacin and cycloserine clustered together (*SI Appendix*, Fig. S6*B*). Taken together, these data suggest that the similarity between cycloserine and moxifloxacin morphological profiles arises from an off-target cell wall-damaging effect of moxifloxacin and inhibition of cell wall synthesis by cycloserine.

The second unexpected profile was from bedaquiline, an ATP synthesis inhibitor, which mapped to cell wall-acting antibiotics ethambutol and imipenem (Fig. 3*A*, white star, and Fig. 3*B*). Components of the mycobacterial cell wall, in particular peptidoglycan (PG) and arabinogalactan (AG), are linked to energy production in the cell with components of glycolysis feeding directly into the synthesis of PG and AG (37, 38). In standard laboratory nutrient-replete medium, the presence of sugars allows Mtb to generate ATP from both glycolysis and TCA cycle through substrate-level phosphorylation and oxidative phosphorylation via the electron transport chain (6). Treatment with bedaquiline shuts down the ability of Mtb to carry out oxidative phosphorylation (6), initiating an energy crisis in which Mtb becomes reliant on substrate-level phosphorylation for ATP generation. We hypothesized that bedaquiline disturbed metabolism in a manner that prevents the synthesis of new PG and AG leading to a morphological profile that resembles cells treated with inhibitors of the cell wall. If our hypothesis is true, we reasoned that bedaquiline should not profile with cell wall-acting antibacterials when grown in media containing a fatty acid as its sole carbon source. We tested this hypothesis by comparing profiles of Mtb grown in standard rich growth medium or a growth medium with the fatty acid butyrate as the sole carbon source. We observed that morphological profiles are highly dependent on growth environment (*SI Appendix*, Fig. S8*A*) with bedaquiline profiles resembling those from cell wall-acting antibacterials only when Mtb is grown in rich medium (*SI Appendix*, Fig. S8*B*). These data support previous observations (6) that the mechanism of action of bedaquiline is dependent on metabolic state of Mtb.

### MorphEUS Correctly Classifies Cellular Targets of Unknown Drugs.

Classification of morphological profiles using MorphEUS shows that distinctive morphological patterns are induced in Mtb according to the terminal stress pathway, which may be the canonical pathway of action or proximal (downstream) effector, as in the case of moxifloxacin and bedaquiline. Because some downstream or off-target effects may be induced at high-dose treatments (or likewise not overshadowed by other pathways at low dose), dose dependencies may be another indicator of noncanonical effects. In support of this hypothesis, we observed strong dose dependencies with morphological profiles of bedaquiline and moxifloxacin (*SI Appendix*, Fig. S9).

**Fig. 4.**
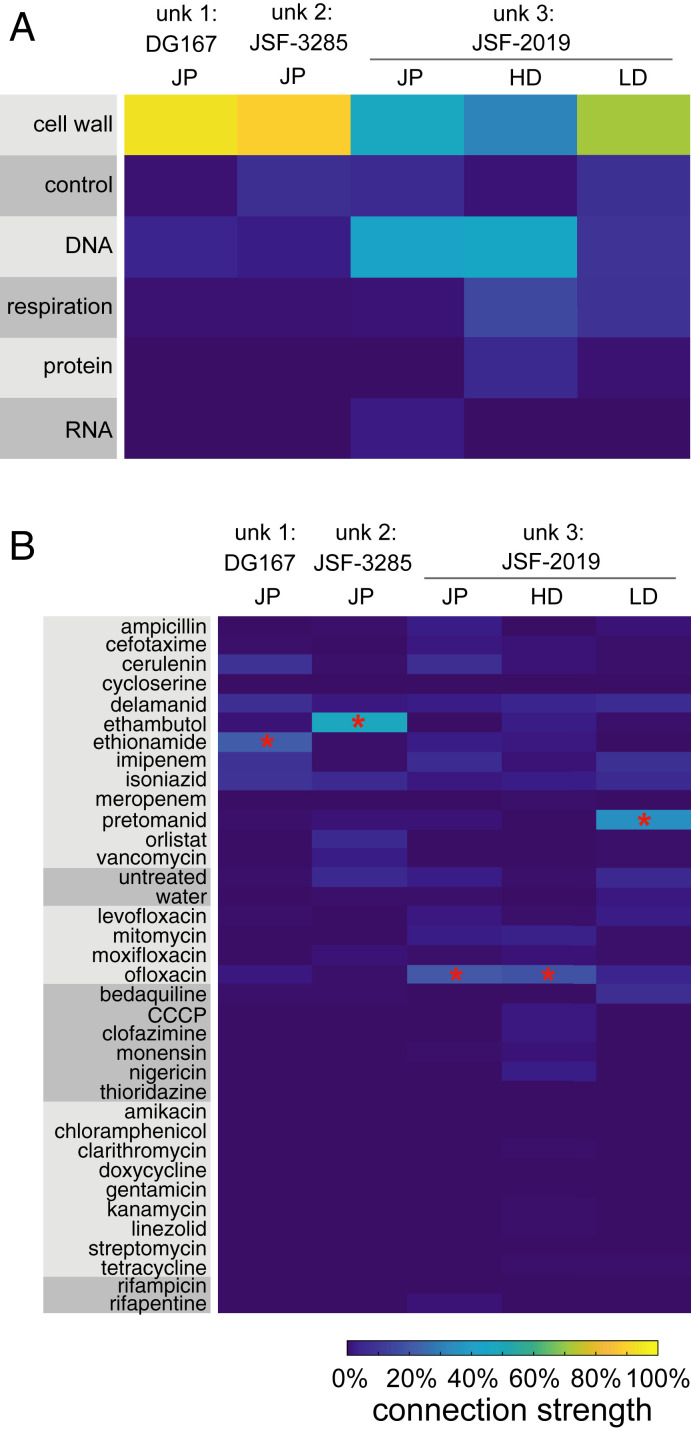
MorphEUS accurately predicts pathways of action of compounds when blinded to mechanism of action. (*A*) cKNN profiles of broad drug categories and (*B*) individual drugs for compounds with anti-TB activities (unk, unknown). Each column corresponds to a different compound or treatment dose. From left to right, joint dose profile (JP) for DG167, JSF-3825, JSF-2019; high dose profile (HD) for JSF-2019; and low dose (LD) profile for JSF-2019: *n* = 7,300, 7,160, 7,742, 7,742, 5,150, and 2,592 respectively. The most similar drug for each MorphEUS classification is indicated by the red asterisk.

Blinded to compound identity, we next used MorphEUS to identify pathways of action for three noncommercial antituberculars with known mechanisms of action. We mapped unknown compounds 1 and 2 as cell wall acting; compounds 1 and 2 were nearest neighbors to ethionamide and ethambutol, respectively (Fig. 4). We unblinded the compound identities to compare their known mechanisms of action to those predicted by MorphEUS. These compounds (DG167 and its derivative JSF-3285) were validated through extensive biophysical, X-ray crystallographic, biochemical binding, and spontaneous drug-resistant mutant studies to be inhibitors of cell wall mycolate biosynthesis through specific engagement of the α-ketoacyl synthase KasA (39, 40). Taken together, our analysis of DG167 and JSF-3285 using MorphEUS has independently validated the target pathway of these two compounds and shown these analogs act through the same pathway of action.

The activity of the third unknown compound was harder to interpret. Unknown compound 3 categorized with both cell wall- and DNA-acting antibacterials by MorphEUS with ofloxacin as its nearest neighbor (via joint dose profiles; Fig. 4). In contrast, MorphEUS analysis at low treatment dose mapped unknown compound 3 to cell wall-acting antibacterials with pretomanid as its nearest neighbor. The dose-dependent effects of unknown compound 3 suggest that downstream off-target effects are amplified with increasing treatment dose. We unblinded the compound to learn if our conclusions were corroborated with previous mechanistic studies performed. Unknown compound 3 is JSF-2019, a triazine that resembles pretomanid in both its F420-dependent production of NO• and its ability to inhibit mycolic acid synthesis, albeit at a different step in the pathway (41). The mechanistic similarity of JSF-2019 and pretomanid validated the MorphEUS prediction of JSF-2019 acting like pretomanid at low dose but did not provide insight into the MorphEUS prediction of DNA targeting activity at high dose.

We hypothesized that the production of NO• by JSF-2019 at high doses induces DNA damage through DNA alkylation (42) in addition to its known cell wall-targeting activity (41). To test if JSF-2019 perturbs DNA processing pathways, we evaluated transcriptional profiles for ofloxacin- and JSF-2019–treated Mtb and found enrichment of coregulated genes involved in DNA damage (43) as well as nucleotide metabolism and biosynthesis (*SI Appendix*, Fig. S10 and Dataset S2). These results indicate that Mtb experiences DNA damage when treated with JSF-2019. A detailed analysis of JSF-2019 resistant mutants (41) uncovered the presence of mutations in *rv2983* and *rv2623* (44–46). Mutations in *rv2983* have previously been found to generate resistance to fluoroquinolones (44) while overexpression of Rv2623 has been linked to exposure of Mtb to ofloxacin or moxifloxacin (45, 46). Together these analyses are supportive that JSF-2019 treatment of Mtb results in damage to the cell wall at low doses and additional damage to DNA at high doses (*SI Appendix*, Fig. S5). This dual activity is easier to recognize in the general categorizations (rather than the drug-specific MorphEUS mapping) because of the relatively strong connection of JSF-2019 to ofloxacin compared to more modest similarities to many cell wall-acting drugs (Fig. 4). We conclude that MorphEUS has enabled us to efficiently focus the analysis of resistance and transcriptomic data to define critical downstream effects that contribute to the mechanism of JSF-2019.

## Discussion

The morphological response of Mtb to drug treatment was subtle and confounded by high levels of heterogeneity in contrast to other bacterial species such as *E. coli*, *B. subtilis*, and *M. smegmatis* (8, 9, 47). Consequently, we could not cluster drug response profiles through a traditional cytological profiling approach (8). We designed MorphEUS to overcome these challenges and enable rapid characterization of drug pathways of action by cellular damage as manifested in physical changes such as cell shape, permeability, and organization. While the changes in features were subtle, they were still drug specific and dose dependent. We found that drugs of the same category influenced the heterogeneity in Mtb’s morphological response in a similar manner, providing us with a valuable set of morphological descriptors to link cellular damage to drug action. To overcome feature subtlety, MorphEUS utilizes the consensus of drug profile relationships from multiple k-nearest neighbor analyses. Here we have optimized the analysis pipeline for Mtb, but incorporation of feature heterogeneity, batch normalization, and consensus profiling may be utilized to achieve morphological profiling in other pathogens and cell types.

In applying MorphEUS to a set of 34 known antibiotics and three blinded, noncommercial antibacterials, we found that MorphEUS grouped antibacterials by their pathways of action, which may be the primary target pathway or off-target effects. Most of the antibacterials profiled with their direct target pathway, but we identified three antituberculars (moxifloxacin, bedaquiline, and JSF-2019) that profiled by their off-target or secondary effects. Bedaquiline and moxifloxacin are known as inhibitors of respiration and DNA synthesis, respectively, yet when MorphEUS was applied, both clustered with cell wall-inhibiting drugs. For bedaquiline, we found that the apparent cell wall damage was specific to metabolic state and appears to be a downstream consequence of Mtb needing to carry out glycolysis for ATP production in the absence of oxidative phosphorylation. This clustering was not observed for cells utilizing the fatty acid butyrate as a sole carbon source, supporting previous studies that show the susceptibility of Mtb to bedaquiline is dependent on metabolic state (6). Understanding drug action in the context of metabolic state is key to developing new TB drug regimens. This is because the treatment must target Mtb within different lesion types, which differ in available nutrients and stressors that alter Mtb state (48–50). An understanding of how drugs kill Mtb in the context of their microenvironment would help us rationally design combination therapies.

There is increasing evidence that polypharmacologies significantly contribute to the bactericidal activity of a drug (4, 11–13). For example, recent reports in *Mycobacterium abscessus* and *Mycobacterium bovis* have shown that treatment with cell wall-acting compounds lead to toxic intracellular accumulations of ATP—a downstream effect that is independent of the cell wall activity of the drug (11, 12). Another example of the bactericidal activity from an off-target effect can be found in *E. coli* with its production of toxic free radicals following treatment with inhibitors of protein, DNA, and cell wall synthesis (4). Having a tool that can rapidly identify such polypharmacologies would be useful in identifying off-target effects that can potentially antagonize another compound when used in combination, such as bedaquiline decreasing the bactericidal ATP burst of isoniazid (12, 13). In the process of applying MorphEUS, we learned that this pipeline allows for hypothesis generation that can be complemented by transcriptomic and genetic approaches by focusing the evaluation of these large systematic datasets. In particular, MorphEUS directed the analysis of transcriptomic data to discover off-target effects of moxifloxacin and JSF-2019 that were not readily apparent from transcriptional profiling alone. These findings highlight the ability of MorphEUS to guide the analysis of other profiling platforms and identify cellular damage incurred beyond primary drug target engagement.

MorphEUS profiling, like all cytological profiling techniques, is data-driven and based on classification among a pool of other profiles. MorphEUS is sensitive to both the breadth and depth of the antibacterials used to create profiles. A limitation of the method is that it requires multiple representative profiles from Mtb treated with compounds known to target the same broad cellular target. For example, our analysis included only two drugs that target RNA polymerase. These drug treatments performed poorly in cross validation, likely as a result of their small class size. We expect the accuracy and resolution of MorphEUS to improve as the drug set is expanded. MorphEUS is also limited in its ability to identify compounds with novel mechanisms of action that are unlike the other profiled drugs. One way to potentially overcome these issues with class representation and identification of novel mechanisms of action would be to increase the breadth of the MorphEUS training set with morphological profiles from genetic knockdown (hypomorph) libraries of Mtb (51). A recent study by de Wet and colleagues (47) created a compendium of morphological profiles in an *M. smegmatis* CRISPRi knockdown library. This study demonstrated that a systematic morphological landscape of essential gene function could be used to link drug treatment profiles to a target pathway (47). Performing MorphEUS on genetic knockdown libraries in Mtb could therefore act as a way to increase the breadth of pathways that can be classified from morphological profiles in a way that is not obtainable by drug treatment alone. Similarly, morphological profiling of drug-resistant mutants of Mtb may provide insight into the mechanisms underlying drug activity by allowing for the separation of primary drug target and drug response due to the agent used (proximal response). These data would then allow for the identification of compounds that target novel pathways in Mtb through the generation of morphological signatures characteristic of genetic knockdown strains belonging to pathways with no known drug targets.

We have shown that the MorphEUS pipeline identifies drug pathways of action in Mtb and also reveals off-target and downstream drug effects that are proximal to antibacterial action. We anticipate that application of MorphEUS to new compounds will reveal polypharmacologies and detail cellular pathways involved from drug engagement to cell death, consequently accelerating the drug development pipeline for tuberculosis. Furthermore, we expect that the success of MorphEUS in profiling drug action in an organism like Mtb with significant inherent heterogeneity and subtle cytological responsiveness indicates the pipeline’s translatability to other pathogens and cell types.

## Materials and Methods

### Bacterial Strains.

Mtb strain used in this study was Erdman. *M. smegmatis* strain used in this study was derived from mc^2^155. *E. coli* strains used in this study were derived from DH5α.

### Growth Conditions.

Mtb cells were cultured in standard medium consisting of 7H9 broth (ThermoFisher; DF0713-17-9) with 0.05% Tween-80 (ThermoFisher; BP338-500), 0.2% glycerol (ThermoFisher; G33-1), and 10% Middlebrook OADC (ThermoFisher; B12351). Frozen 1 mL Mtb stocks were added to 10 mL of standard medium and grown with mild agitation in a 37 °C incubator until the culture reached an OD_600_ of ∼0.4 to 0.7. The bacteria were then subcultured into 10 mL of fresh medium to an OD_600_ of 0.05 and grown to an OD_600_ of ∼0.4–0.7. At this time the cells were plated onto 96-well plates containing drugs at the predetermined amounts (see below). Drug-treated plates were incubated at 37 °C in humidified bags until fixation.

Mtb cells were adapted to low-pH standard medium by first growing and subculturing the cells once in standard medium (as described above) followed by centrifugation and resuspension in standard medium supplemented with 100 mM 2-(*N*-Morpholino)ethanesulfonic acid hydrate (SigmaAldrich; M2933) HCL adjusted to pH of 5.8. Cells were subcultured once in low-pH standard medium before plating.

Mtb cells grown with butyrate or cholesterol as their sole carbon source were cultured in base medium (7H9 broth with 0.05% Tyloxapol, 0.5 g/L Fatty Acid-free BSA, 100 mM NaCl, 100 mM Mops buffer [SigmaAldrich; M3183], and HCL adjusted to pH 7.0) supplemented with either 10 mM sodium butyrate (SigmaAldrich; 303410) or 0.2 mM cholesterol (SigmaAldrich; C8667). Sodium butyrate was added directly to the base medium while cholesterol was dissolved in a 50/50 (vol/vol) mixture of tyloxapol and ethanol to obtain a 100 mM stock solution as previously described (52). Bacteria grown in butyrate medium were grown and subcultured once in standard medium before centrifugation and resuspension in butyrate medium. The cells were subcultured once using fresh butyrate medium before they were aliquoted into tubes (1 mL each) which were stored at −80 °C until use. Frozen stocks were started and subcultured in butyrate medium before plating. Bacteria grown in cholesterol medium were grown and subcultured once in standard medium before centrifugation and resuspension in cholesterol medium to an OD_600_ of ∼0.2. The bacteria were plated upon reaching an OD_600_ of ∼0.4.

*M. smegmatis* cells were cultured in standard medium supplemented with Middlebrook ADC (ThermoFisher; B12352); 100 μL frozen stocks were added to 10 mL of standard ADC medium and subcultured once before use. *E. coli* cells harboring plasmids used in this study were grown in LB broth containing appropriate antibiotics (50 μg/mL hygromycin or 25 μg/mL kanamycin).

### Drug Treatments.

For time-dose–response profiling, drugs were loaded into 96-well plates with the HP D300e digital drug dispenser. Each drug used in the study was reconstituted, depending on drug solubility, in water, DMSO, 1 N NaOH, or methanol solubility at a concentration between 2.5 and 100 mg/mL (*SI Appendix*, Table S1). Reconstituted drugs were then aliquoted in single-use sterile tubes and stored at −20 °C until use. The percentage of DMSO for all drug treatments was between 0.00045 and 0.75% except for ampicillin, tetracycline, chloramphenicol, and thioridazine high-dose treatments, where DMSO percentage did not exceed 1.5%. Even at 1.5% DMSO, profiles of untreated cells and DMSO-treated cells were indistinguishable. To determine whether solvents elicited morphological changes that would impact the profiling, we tested whether cells treated with each control condition (DMSO at different concentrations, and the highest levels used in the other solvents: 0.3% 1 N NaOH, 0.1% methanol, or 3% water) profiles with each other and untreated samples, which would suggest that the solvents were not drivers of morphological changes. We selected a feature set based on the high-dose MorphEUS analysis, keeping features that were used in over half of the classification trials, resulting in a set of 28 features. Using the untreated profiles from the high-dose analysis, we performed typical variation normalization (TVN) on the range of DMSO treatments followed by PCA. We searched for the first nearest neighbor for each individual treatment to see if the same treatment groups were nearest neighbors with each other. We found no likeness (e.g., 100% confusion) between the same treatment groups (e.g., that the controls were not identifiable into similar treatment groups), suggesting that solvents alone did not induce strong morphological effects.

### IC90 Determination.

Mtb and *M. smegmatis* cultures were grown from frozen aliquots and subcultured once as described above. Once grown to an OD_600_ of ∼0.4 to 0.7, the cells were diluted to an OD_600_ of 0.05 and added to 96-well plates containing drugs in a twofold dilution series for nine concentrations. Each treatment series contained an untreated well as a control. All IC90 determinations were performed in biological triplicate. To avoid plate effects, wells around the perimeter of the plate were not used. An initial OD_600_ plate read was performed immediately for each *M. smegmatis* plate, while for Mtb cultures this was performed after allowing the bacterial cells to settle overnight. A second plate read was performed for *M. smegmatis* after 24 h and Mtb after 5 d of incubation. Growth inhibition curves were generated by subtracting the initial reads from the final reads and then normalizing the data to untreated controls. The IC90 was defined as the drug concentration that inhibited at least 90% of all bacterial growth.

### Fixation of Antibiotic-Treated Mtb-Bacilli.

After the designated treatment times (overnight unless otherwise noted), Mtb cultures were fixed in paraformaldehyde (Alfa Aesar; 43368) at a final concentration of 4% and transferred to clean 96-well plates. The plate was surface decontaminated with vesphene IISE (Fisher Scientific; 1441511) and sealed with Microseal F foil seals (Biorad; MSF1001). The duration of fixation was 1 h total. After fixation, the cells were washed twice with 100 µL of PBS (ThermoFisher; 20012-027) + 0.2% Tween-80 (PBST), then resuspended in 100 μL of PBST, sealed (ThermoFisher optically clear plate seals, AB1170) and stored at 4 °C until staining and imaging.

### Staining and Fluorescent Imaging of Mtb Cells.

All staining was performed in 96-well plates with 50 µL of fixed Mtb cells diluted in 50 µL of PBST. Staining was performed with 0.6 μg of FM4-64FX (ThermoFisher; F34653) and 15 μL of a 0.1 μM SYTO 24 (ThermoFisher; S7559) stock in each well containing PBST and fixed bacilli. The plate was then incubated at room temperature in the dark for 30 min. Once stained, the cells were washed once with an equal volume of PBST and resuspended in 30 µL of PBST. Stained Mtb were spotted onto agar pads (1% wt/vol agarose; SigmaAldrich; A3643-25G). Images were captured with a widefield DeltaVision PersonalDV (Applied Precisions) microscope. Bacteria were illuminated using an InsightSSI Solid State Illumination system with transmitted light for phase contrast microscopy. SYTO 24 was imaged using Ex. 475 nm and Em. 525 nm. FM4-64FX was imaged with Ex. 475 nm and Em. 679 nm. Montage images were generated using a custom macro that captures 25 individual fields of view per image. Two technical replicate images were taken from each sample for a total of 50 images per biological replicate. Three biological replicates were generated for each drug treatment. Images were recorded with a DV Elite CMOS camera for all three channels.

### Generation of RpoB-EGFP Strain.

A strain of *rpoB-egfp* in the *M. smegmatis* mc^2^155 background was generated using the ORBIT recombineering system developed by Murphy et al. (53). Briefly, a frozen aliquot of *M. smegmatis* was grown and subcultured once as described above. Upon reaching midlog phase, the cells were washed twice with 10% glycerol (Fisher Scientific; G33-1) and electroporated with pKM444. The plasmid pKM444 allows for ATC inducible expression of Che9c RecT and Bxb1 integrase phage proteins and harbors a kanamycin resistance cassette. Transformants were selected for on Middlebrook 7H10 plates (ThermoFisher; BD 2627) with ADC containing 25 μg/mL of kanamycin (VWR; 0408-10G). A control without plasmid was also plated to ensure proper kanamycin selection. The pKM444 harboring strain of *M. smegmatis* was then grown to an OD_600_ of 0.5 in standard ADC medium containing 25 μg/mL of kanamycin. Once the desired OD_600_ was reached, anhydrotetracycline (ATC; Fisher Scientific; 13803-65-1) was added, and the cells were incubated with gentle agitation until an OD_600_ of 0.8 was reached. The cells were then washed with glycerol as described above and electroporated with 1 μg of a *rpoB* targeting oligo harboring an *attP* sequence (see below) and 0.2 μg of the nonreplicating *egfp* harboring plasmid pKM468-EGFP. pKM468-EGFP contains an *attB* recombination downstream of the *egfp* gene for C-terminal translational fusions, lacks a mycobacterial origin of replication, and harbors a hygromycin resistance cassette. -Oligo+plasmid and -oligo-plasmid controls were also performed as negative controls. Transformations were recovered in 1 mL of standard ADC medium, incubated for 3 h then plated on 7H10-ADC plates containing hygromycin B at 50 μg/mL The presence of the C-terminal EGFP translational fusion to RpoB was validated by fluorescence microscopy using the FITC (Ex. 475 nm Em. 525 nm) channel as described above. The *rpoB* targeting oligo sequence was 5′-GCA​CGT​AAC​TCC​CTT​TCC​CCT​TGC​GGG​TGT​TGA​AAC​TTG​ACT​ACT​GAG​GCG​GTC​TTC​GGA​CGA​GGC​TCT​AGG​TTT​GTA​CCG​TAC​ACC​ACT​GAG​ACC​GCG​GTG​GTT​GAC​CAG​ACA​AAC​CCG​CGA​GAT​CCT​CGA​CGG​ACG​CGG​ATT​CGT​TGC​GCG​ACA​GGT​TGA​TTC​CCA​GGT​TCG​CGG​CAG​CGC​GCT​CC-3′.

### Live-Cell Microscopy.

*M. smegmatis* cells expressing RpoB-GFP were grown overnight from frozen 100 μL aliquots in 10 mL of fresh standard ADC medium. The bacteria were subcultured once and allowed to reach midlog phase (OD_600_ ∼0.5 to 0.7). The culture was then filtered to remove aggregates of bacteria and loaded into a custom polydimethylsiloxane (PDMS) microfluidic device as previously described (54). Fresh medium was delivered to cells using a microfluidic syringe pump. The microfluidics device was attached to a custom PDMS mixing device for delivery of drug for the duration of time described below and then placed on an automated microscope stage inside an environmental chamber that was maintained at 37 °C. The bacteria were imaged for a total of 26 h using a widefield DeltaVision PersonalDV (Applied Precision, Inc.) with a hardware-based autofocus. Antibacterial compounds were introduced to the *M. smegmatis* after a 10-h growth phase. Drug treatment lasted for 6 h and was followed by a 10-h recovery phase.

### Transcriptional Profile Analysis.

The transcriptional profiles of JSF-2019, bedaquiline, and moxifloxacin were obtained from GSE126718 (41), GSE43749 (6), and GSE71200 (26), respectively. The transcriptional profiles of other compounds were extracted from GSE1642 (27). Genes involved in the Mtb SOS response were defined as genes of the *lexA* and *recA* regulons (30–32). Genes involved in cell wall damage and repair were defined as the 3 genes of the *iniBAC* operon involved in addition to the 38 genes within peptidoglycan biosynthesis (33, 35, 36). The 166 genes which were significantly coup-regulated and codown-regulated by both JSF-2019 and ofloxacin (judged by fold change >1.5 and FDR *P* < 0.01) were selected from the transcriptional profile (Dataset S2). *rv0560c*, which encodes a benzoquinone methyltransferase involved in xenobiotics metabolic detoxification (55, 56), was removed from the list due to its extremely high induction fold in JSF-2019 rather than in other compound treatments. A hierarchical clustering analysis was applied to the 165 genes according to Pearson correlation via the R packages pheatmap and ggplot. The function and pathway enrichment analysis of the 155 genes were performed via a gene ontology resource (geneontology.org/).

### UMAP.

To implement the UMAP dimensionality reduction algorithm (47, 57), we used the umap package in R (https://cran.r-project.org/web/packages/umap/index.html). We used the umap function contained in this package and exported the results to a CSV file to be plotted in MATLAB.

### MorphEUS Analysis Pipeline.

Overview of Mtb morphological profiling analysis (MorphEUS). The MorphEUS analysis pipeline (Fig. 2) is as follows.

#### Feature quantification.

1) Single-cell measurements extracted from MicrobeJ are imported to MATLAB to 2) undergo quality control before bulk analysis. 3) Variables describing the cell-to-cell variation within each feature are calculated prior to feature selection and profile classification.

#### Classification trial.

4) Nonredundant features are iteratively selected. 5) Normalization is performed across replicates to decrease experimental noise (TVN). 6) PCA is performed on the batch-normalized, reduced feature dataset and followed by 7) k-nearest neighbors (KNN) analysis on the PCA scores matrix. KNN analysis classifies drugs (e.g., treatment groups) by identifying the nearest neighbors (most similar profiles). These nearest neighbors are represented as a matrix or a network diagram (graph).

#### Classification consensus (consensus KNN).

To overcome the fragility of feature selection, we generate a consensus of multiple (70) classification trials, wherein each trial utilizes a different, stochastically selected set of 80 untreated samples for feature selection and batch normalization. The resulting consensus KNN (cKNN) may be visualized using a network diagram where edges between drugs are color-coded according to how often their profiles were nearest neighbors (% of total trials) or a matrix that describes the frequency of drug–drug links among trials (Fig. 3). The most similar drug profiles are linked in a large number of the trials. The connectivity maps and corresponding summary heat maps are used to make informed predictions about the target pathway of an antibiotic.

### Image Segmentation and Feature Extraction.

Before image segmentation, we used the ImageJ plugin BaSiC to ensure an even distribution of illumination in all channels across the image (58). Image segmentation was performed using the ImageJ plugin MicrobeJ [v 5.13l (1)], extracting seven features from phase contrast and nine from each fluorescent channel using custom settings, resulting in a total of 25 features (*SI Appendix*, Table S2) (59). The image segmentation in MicrobeJ is computationally demanding and therefore was run on a high-performance computing cluster.

### Blur Thresholding.

All data were organized and analyzed using custom scripts in MATLAB 2019a. Out-of-focus bacilli were identified from the transverse phase-contrast profile of each bacterium and discarded. The profile of an in-focus, well-segmented bacillus has a Gaussian distribution with high intensity around the edge of the bacterium, followed by a steep drop after the edge. Blurry cells were filtered out with the following criteria: goodness of fit to a Gaussian distribution, if the minimum point was off center, unexpected local maxima, difference in intensity between minimum and maximum values, difference in intensity between edges, and slope of the edges. With exception to the fluorescent foci counts, the median, first quartile, third quartile, and interquartile range were calculated for each feature to account for the population distribution, resulting in 94 features total. Distribution features were not calculated for foci count features because these measurements are discrete, not continuous, features. Features were then normalized, dividing by the largest value for the feature across all treatments.

### Typical Variance Normalization.

TVN aligns the covariance matrices produced by PCA of untreated control data from each experimental plate, or batch, and applies this transformation batch by batch to allow for less biased comparison of the drug-treated cells across plates and replicates (60). An abbreviated version of TVN was applied to reduce batch effects from imaging. First PCA is performed on the untreated controls. Each axis is scaled to have a mean of zero with variance of 1. This transformation is then applied to the entire dataset, including treated and untreated samples (*SI Appendix*, Fig. S4). To perform TVN, we dedicated 25% of our samples in every experiment and imaging session to be untreated controls. Each classification trial begins with stochastically selecting 80 untreated controls; thus, feature sets were restricted to a maximum of 79 features since a PCA transform with *n* samples can only have *n* − 1 features.

### PCA.

PCA was performed on the normalized data (feature selected or not, as indicated) using the built-in MATLAB function. In the BCP pipeline, PCA reduces the dimensionality of the data, allowing variance across all of the features to be visualized in three or fewer dimensions. After accounting for heterogeneity with batch normalization and including features of variation into the profiles, some drug clustering was observed, especially among cell wall-acting antibacterials (*SI Appendix*, Fig. S2 *A*, *Lower*). By PCA, bedaquiline clustered with cell wall-acting drugs in standard, rich growth medium (*SI Appendix*, Figs. S2 *A*, *Lower*, and S8 *B*, *Left*) but not in fatty acid-rich growth medium (*SI Appendix*, Fig. S8 *B*, *Right*).

### KNN.

KNN analysis was implemented using the cosine distance metric and the *knnsearch* MATLAB function. K was set to 1; thus, only the first nearest neighbor was identified. For our setup, we took the median PCA score from the three replicates for each drug as inputs for the KNN analysis. The KNN algorithm finds the k-nearest neighboring points where the cosine distance between PCA scores is shortest. MATLAB defines the cosine distance as 1 minus the cosine of the included angle between points. We observed that feature selection was dependent on which untreated samples were included in the TVN batch-to-batch normalization process (80 from 117), suggesting there are many good solutions or feature sets that can lead to similar profiling of the drug target. To ensure our classification was not overfitting the data depending on which untreated samples were included in the analysis, we took a stochastic approach. We define the application of PCA and then KNN analysis on a particular set of reduced features as a classification trial. The MorphEUS pipeline steps 4 to 7 were repeated for 70 classification trials, each including a different randomly selected set of 80 untreated controls (classifications converged by 70 trials; *SI Appendix*, Fig. S9).

### Iterative Feature Selection.

To reduce overfitting and noise in our 94-variable feature set, we utilized the minimum redundancy maximum relevance (mRMR) feature selection algorithm (61). Here we customized previously published MATLAB code to perform mRMR feature selection using the mutual information difference scheme (61). Because the algorithm rank orders variables and does not automate the selection of the optimal number of features, we implemented an iterative feature selection method that rewards runs that result in more drug–drug connections with same target pathways (*SI Appendix*, Table S1). Since we begin with 94 features but are limited to 79 variables by our TVN analysis, mRMR was used to rank order the top 79 features. Starting with the 79 rank-ordered features, we removed each feature individually and performed TVN, PCA, and KNN analysis on the remaining feature set. Success of the feature set was quantified by accuracy of the KNN in linking drugs belonging to the same broad category assigned by literature review (*SI Appendix*, Table S1). The feature set that resulted in greatest model accuracy was selected, and the variable removal process was repeated until maximal prediction performance was reached. On average, these iteratively determined feature sets contained 38 variables.

### cKNN.

The cKNN results compiled from all 70 classification trials were visualized using a network map and heat map, where edge color and grid square color, respectively, correspond to how frequently two drug profiles were identified as nearest neighbors. Because our goal was to identify similar treatment profiles, connections between profiles in the cKNN were made undirected, and the drug–drug categorization matrices (such as Fig. 3 *B*, *Left*) are symmetric. These visuals allow for classification of the target biological pathway of each drug based on the fact that the robustness of phenotypic similarities between drug profiles can easily be evaluated. All maps and plots were generated in MATLAB 2019a.

### Comparison to Random.

We evaluated model performance by testing how accurate MorphEUS was when the drug categories were randomly assigned. To do so, the labels for the drugs in the final cKNN were randomly swapped, resulting in 22% accuracy compared to 94% for the joint dose MorphEUS analysis.

### Cross Validation and Classification of Unknown Compounds.

To test the strength of our model, we performed cross validation. This was done by removing 1 of the drugs out of our 34-drug set and running the remaining 33 drugs through the MorphEUS pipeline. The PCA transformation created by the 33 drugs was applied to the TVN-normalized, removed drug, and KNN analysis was performed. At the end of the 70 trials, a cKNN was created, and the pathway of action of the cross-validated drug was classified in accordance to its strongest drug connections and their corresponding pathway(s) of action as classified in *SI Appendix*, Table S1.

### Low Dose, High Dose, and Joint Dose Profiles.

Mtb cytological features are dependent on drug target but also treatment dose and duration (*SI Appendix*, Fig. S6). This raised the possibility that morphological profiles from a low dose of treatment or a joint profile of low- (0.25 × IC90) and high- (3 × IC90) dose treatments would improve the accuracy of drug classification using the full drug set and subsequent cross validation. To investigate whether a joint dose profile best describes the variation in the morphological response in Mtb, the full 94 feature datasets from both drug doses were concatenated, resulting in 188 total features. We also applied MorphEUS to low-dose and high-dose treatments as separate profiles. We observed high accuracy using each of the dose treatments (low, high, and joint as 97, 91, and 94%, respectively), but the joint dose profiles were better cross validated (76%) compared to high- (68%) and low- (62%) dose MorphEUS. We therefore use joint dose profiling as the default for MorphEUS.

### Classification of Unknown Compounds.

We apply new compounds to MorphEUS in the same manner as cross validation, only the MorphEUS pipeline is done on the full 34-drug set, and the unknown is added to the set for the final KNN during each classification trial.

### Statistical Analysis.

We performed the Kruskal–Wallis test to identify drug treatments that induce significantly different morphological features compared to untreated cells in rich medium (Fig. 1*A* and *SI Appendix*, Fig. S8A). Through the MorphEUS pipeline utilizes population-based features, the Kruskal–Wallis test was applied to the features of individual cells (*n* = 1,625 to 3,983 for rich, *n* = 1,029 to 6,733 for conditions). The Kruskal–Wallis test was applied to each drug and/or environmental condition individually, per feature. In each case the null hypothesis was that the median feature value for Mtb populations exposed to a specific drug and/or environmental stress was drawn from the same distribution as the median feature value for the untreated controls.

### Data and Materials Availability.

All data needed to evaluate the conclusions in the paper are present in the paper and/or *SI Appendix*. All code for analysis has been deposited at https://gitlab.tufts.edu/aldridgelab-morpheus/morpheus.

## Supplementary Material

Supplementary File

Supplementary File

Supplementary File

Supplementary File

Supplementary File

Supplementary File
