# Supplementary material for: Morphological profiling of tubercle bacilli identifies drug pathways of action
Source: Proc Natl Acad Sci U S A. 2020 Jul 17;117(31):18744–53. doi: 10.1073/pnas.2002738117 (PMC7414088; doi:10.1073/pnas.2002738117)
Supplement: Supplementary File [file pnas.2002738117.sapp.pdf]

Supplementary Information for

Morphological profiling of tubercle bacilli identifies drug pathways of action

Authors: Trever C. Smith II[1,2†], Krista M. Pullen[1,3†], Michaela C. Olson[1], Morgan E. McNellis[1], Ian Richardson[1,4], Sophia Hu[5], Jonah Larkins-Ford[1,6,7], Xin Wang[8], Joel S. Freundlich[8,9], D. Michael Ando[10], and Bree B. Aldridge[1,2,6,7,11*]

Correspondence to:

Bree B. Aldridge

Email: bree.aldridge@tufts.edu

This PDF file includes:

- Figures S1 to S10
- Tables S1 to S2
- Captions for movies S1 to S3
- Captions for datasets S1 and S2

Other supplementary materials for this manuscript include the following:

- Movies S1 to S3
- Still images for movies S1 to S3
- Datasets S1 and S2

www.pnas.org/cgi/doi/10.1073/pnas.2002738117

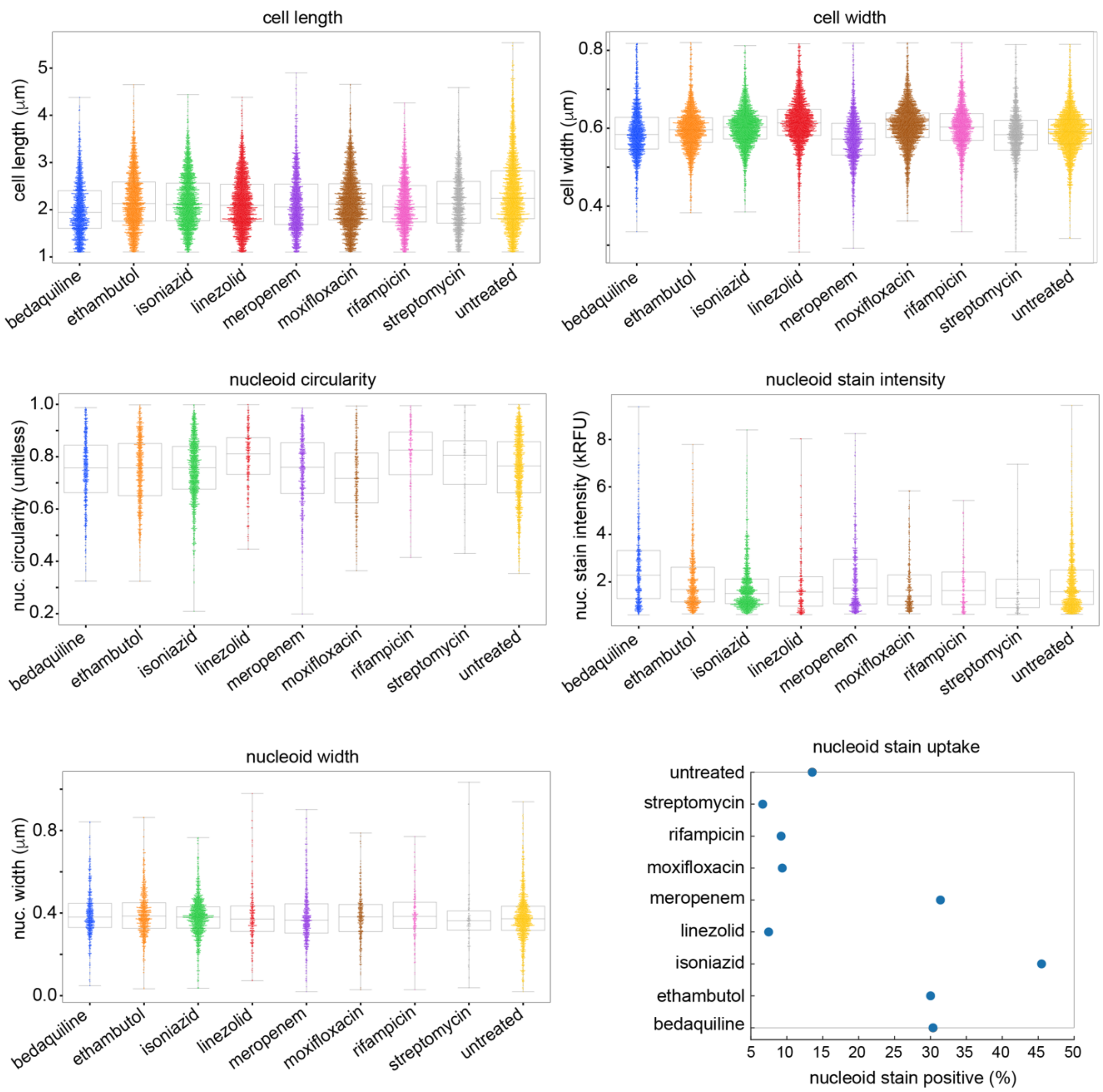

Figure S1. Innate cell-to-cell heterogeneity in Mtb morphologies. Dot and box plots of Mtb morphological features in untreated (yellow) and drug-treated bacilli. Many bacilli are not stained with SYTO 24, therefore only non-zero values are shown for nucleoid features. Cellular heterogeneity is apparent among the most basic cytological features, including cell length, intensity of nucleoid staining, and nucleoid width, even in untreated Mtb (CV=0.31, 0.64, 0.32,

respectively). All plots were generated using the statistical data visualization library Seaborn for Python^{TM}.

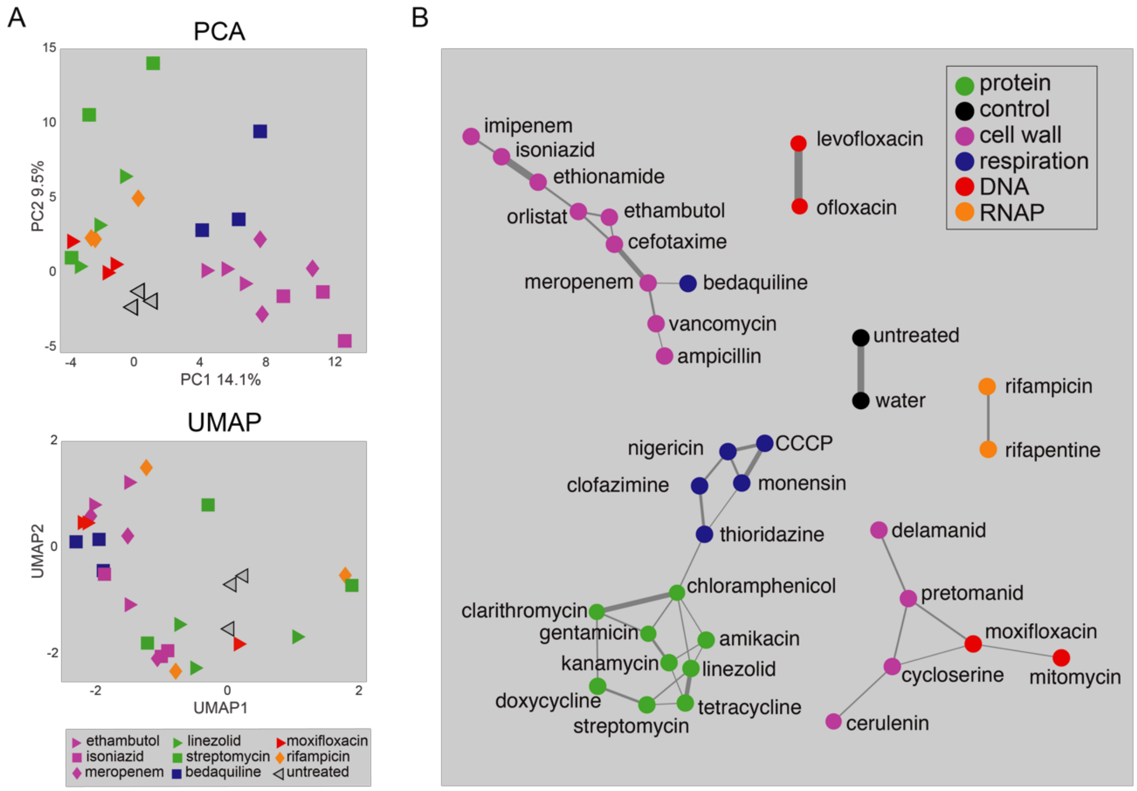

Figure S2. Inherent heterogeneity of Mtb and subtle morphological features requires multistep, multivariable analysis to define cytological profiles. (A) PCA (top) and UMAP (bottom) analyses of eight drug treatments at high dose after incorporating metrics of heterogeneity [quartile 1, quartile 3, and interquartile range (IQR)], feature selection, and batch normalization (TVN; bottom). n=3032-9651 for each treatment group over biological triplicate. TVN aligns the covariance matrices resulting from PCA and whitening of untreated control data from each batch and applies this transformation batch-by-batch to enable comparison across plates and replicates. (B) cKNN connectivity map for high dose drug treatment of Mtb. Drug nodes are colored by broad target category and edge thickness corresponds to how frequently profiles are nearest neighbors in the classification trials (connections are shown for links >12%).

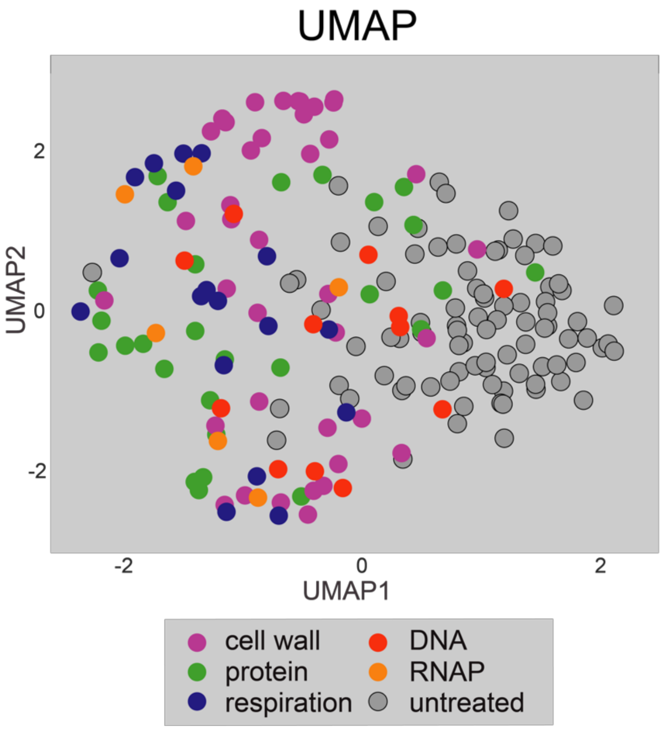

Figure S3. UMAP analysis of 34 drug treated Mtb profiles. Mtb drug profiles from 34 different antibacterials at 3X IC90 (Table S1) after expansion of quantified features (Q1, IQR, Q3) and post TVN. Data points represent individual biological replicates of a drug treatment and are color-coded based on the broad cellular target as determined by literature review (Table S1).

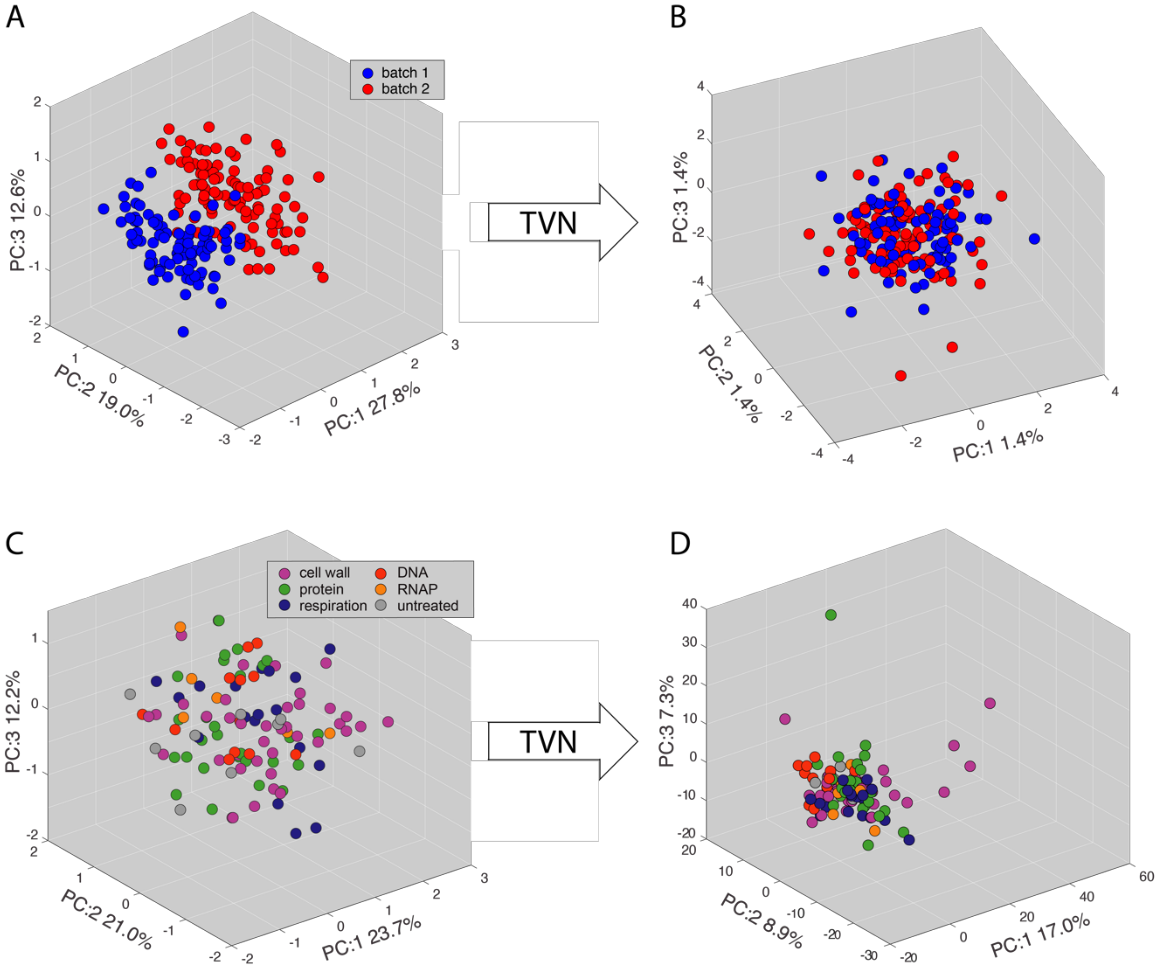

Figure S4. Correction of batch-to-batch heterogeneity. (PCA) of untreated data labeled by batch. (A) PCA prior to TVN normalization. (B) PCA after TVN normalization. PCA of all 34 drug treatments labeled by target pathway using all 94 features. (C) PCA prior to TVN normalization. (D) PCA after TVN normalization. No feature selection was applied.

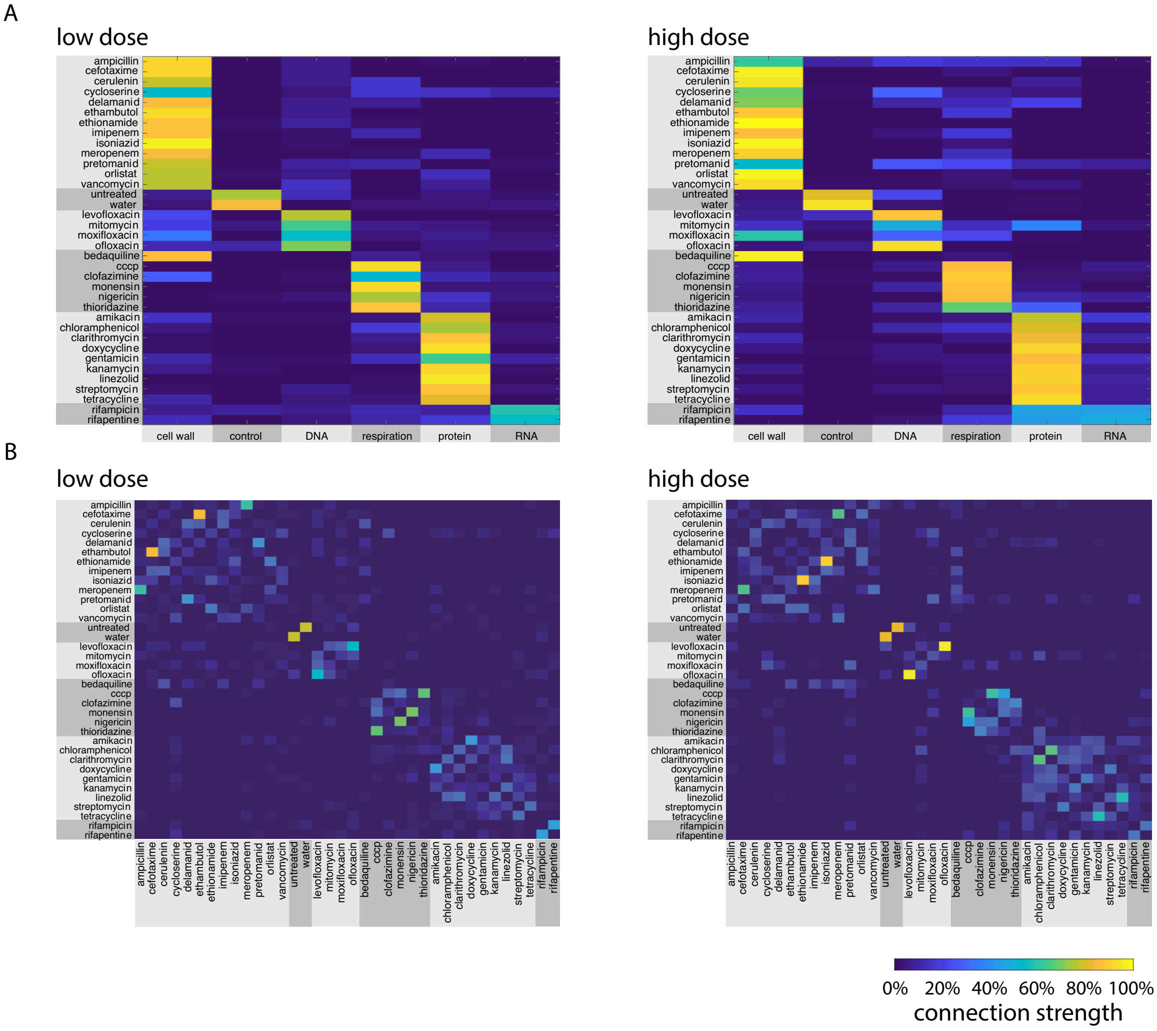

Figure S5. Categorization of drug profiles using MorphEUS. (A) Broad categorization for low dose (left) and high dose (right) profiles are represented as heatmaps. The connection strength is determined by the strength of the consensus k-nearest neighbor analysis.100% strength indicates that the nearest neighbor identified in every iteration was from the same target category while 0% indicates that the drug of interest was never a neighbor to a drug in that pathway. Drugs are listed in the rows and collection of drugs within the broad target pathways are listed in the columns. (B) Heatmaps of drug nearest neighbor pairings for low dose (left) and high dose (right) treatment. The heatmaps are described in Fig. 3. The high dose profiles were 91% accurate for assignment of broad drug category (compared to 26% for randomized categorization) with 68% accurate cross

validation. The low dose profiles were 97% accurate for assignment of broad drug category with 62% accurate cross validation. Pairwise drug connection strength values can be found in Dataset S1 for low (Sheet 2) and high (Sheet 3) dose profiles.

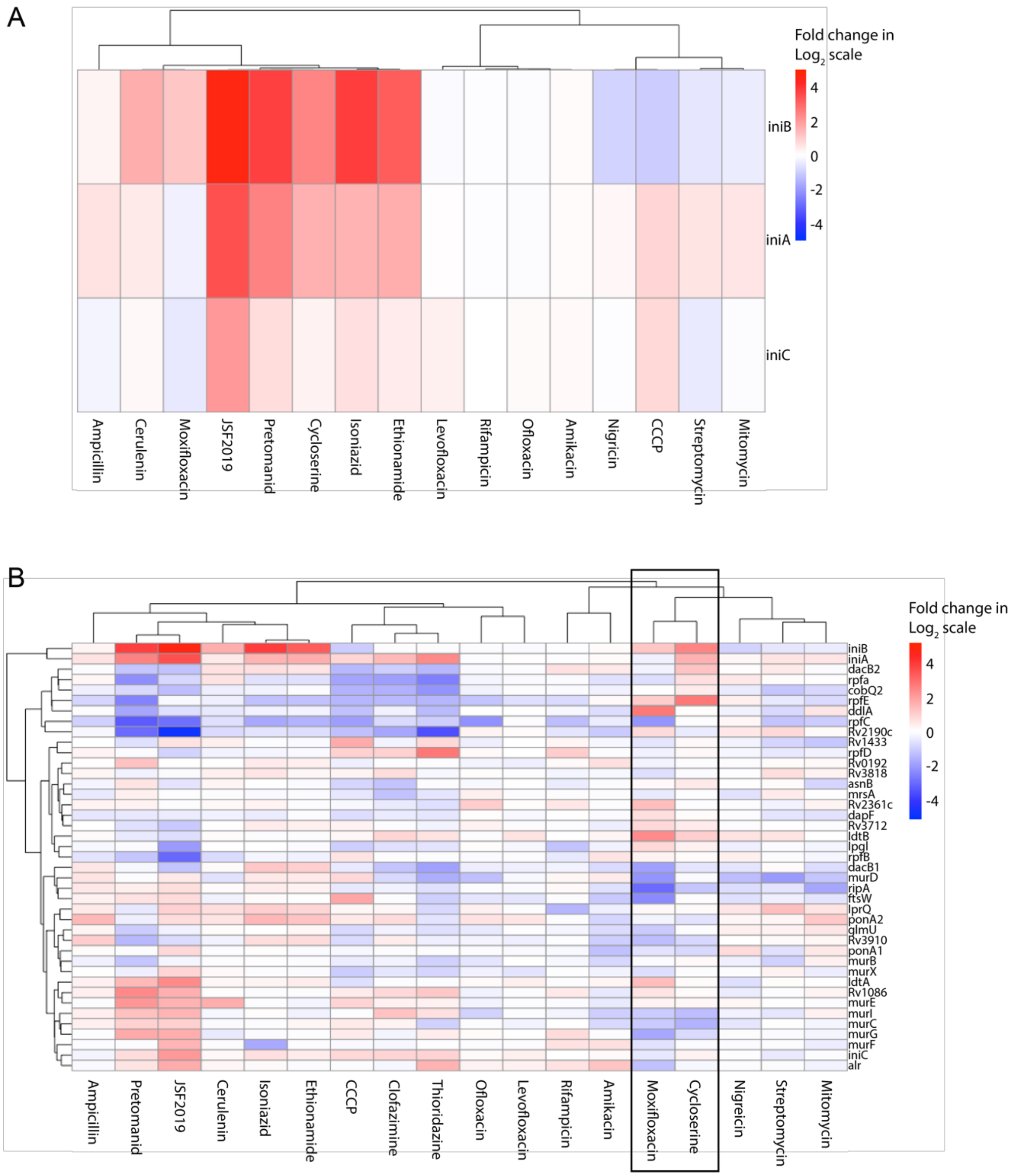

Figure S6. Transcriptional response of Mtb to antibacterials in genes involved in cell wall damage and biosynthesis. (A) Expression profiles of the *iniBAC* operon in response to 16 antibacterials. Known inducers of the *iniBAC* operon isoniazid (INH), ethambutol (ETH), and pretomanid are plotted as controls (B) Expression profiles of genes involved in peptidoglycan

biosynthesis in response to treatment with antibacterials. A box is drawn to highlight the profiles of cycloserine and moxifloxacin, which cluster together. Hierarchical clustering was performed using Pearson distance.

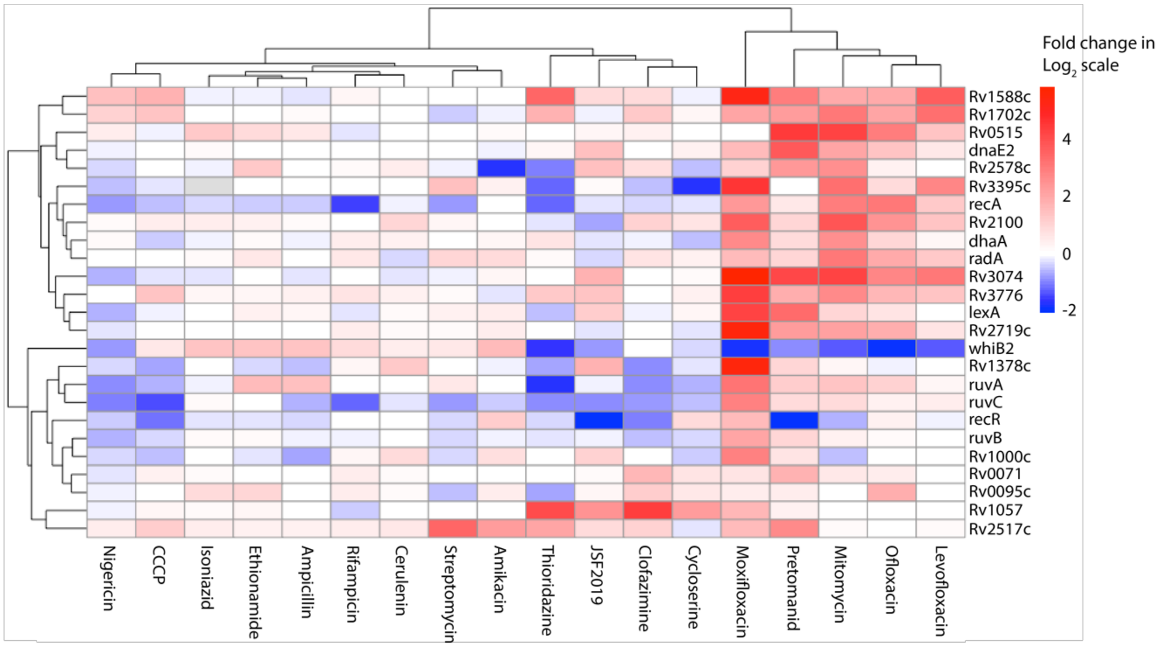

Figure S7. Transcriptional response of Mtb to antibacterials in genes involved in DNA damage. Expression profiles of genes involved in the Mtb SOS response upon treatment with antibacterials. Genes involved in the Mtb SOS response were defined as *recA*-*lexA* regulated genes in Mtb (1-3). Hierarchical clustering was performed using Pearson distance.

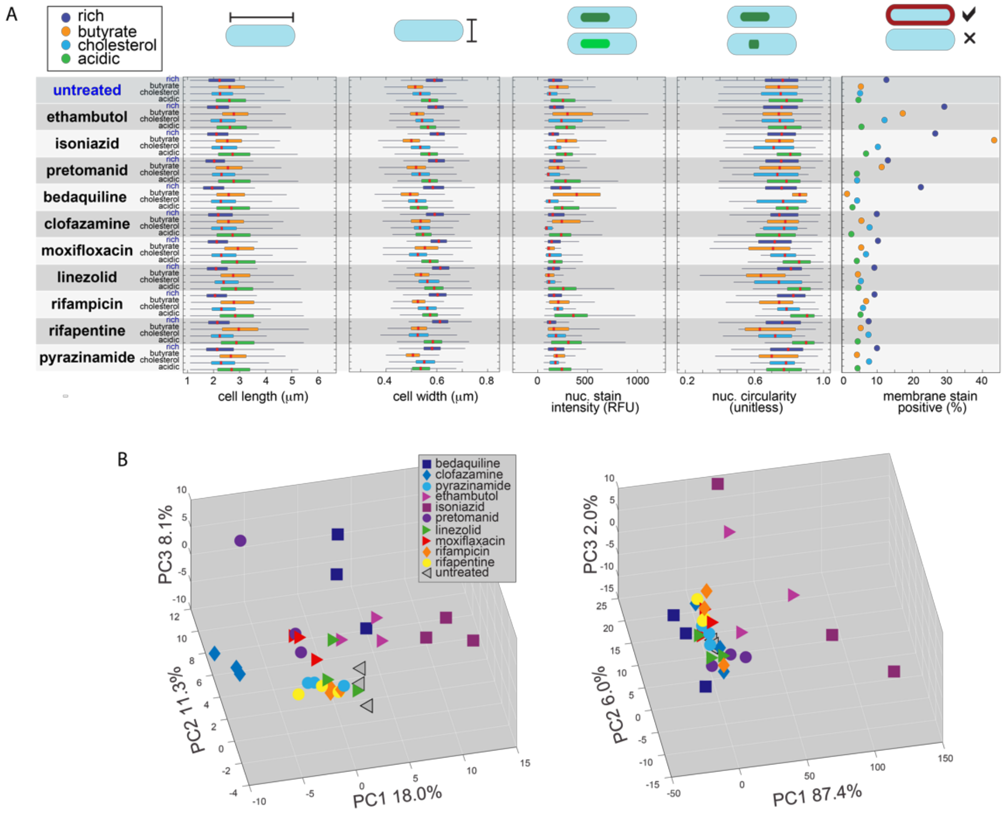

Figure S8. Morphological features and response to drug treatment are dependent on growth conditions. (A) Mtb were treated with each antibiotic (or untreated control) following adaptation to different growth media: rich (blue), butyrate as the sole carbon source (orange), cholesterol as the sole carbon source (cyan), and low pH in rich medium (green) as described in Methods. Red lines mark the medians, boxes mark the 25-75th percentiles, and the whiskers extend the full range, excluding outliers (n=1029-6733). (B) PCA of morphological profiles in Mtb treated with antibiotics. Mtb cells were grown in media containing carbohydrates (left) or butyrate (right) as the sole carbon source. Bedaquiline (dark blue squares) clusters close to the cell wall acting antibiotics (pink and purple shapes) in media containing carbohydrates (left) but not in butyrate (right).

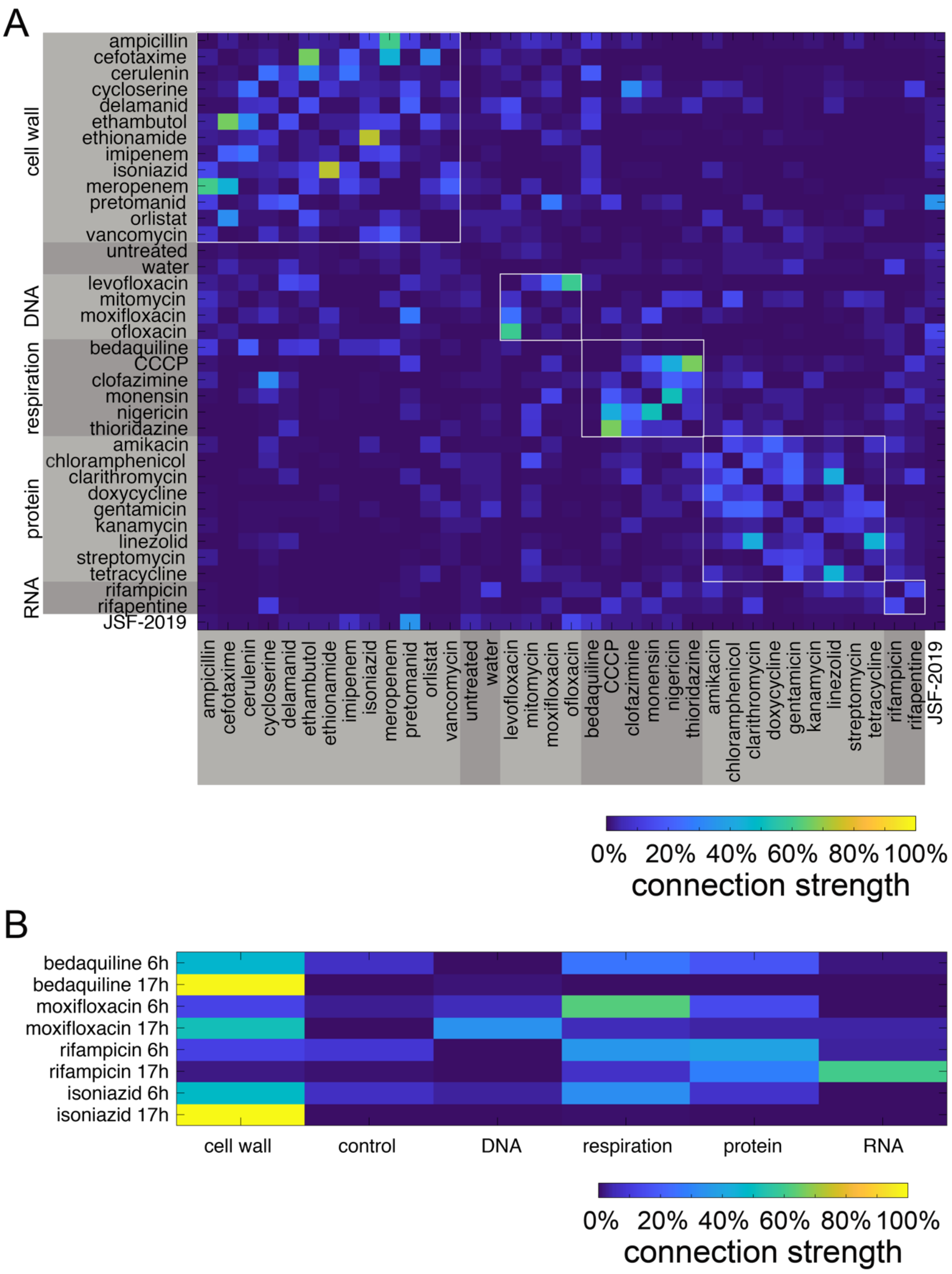
A
cell wall
control
DNA
respiration
protein
RNA
ampicillin
cefotaxime
cerulenin
cycloserine
delamanid
ethambutol
ethionamide
imipenem
isoniazid
meropenem
pretomanid
orlistat
vancomycin
untreated
water
levofloxacin
mitomycin
moxifloxacin
ofloxacin
bedaquiline
CCCP
clofazimine
monensin
nigericin
thioridazine
amikacin
chloramphenicol
clarithromycin
doxycycline
gentamicin
kanamycin
linezolid
streptomycin
tetracycline
rifampicin
rifapentine
JSF-2019
0% 20% 40% 60% 80% 100%
connection strength
B
bedaquiline 6h
bedaquiline 17h
moxifloxacin 6h
moxifloxacin 17h
rifampicin 6h
rifampicin 17h
isoniazid 6h
isoniazid 17h
cell wall
control
DNA
respiration
protein
RNA
0% 20% 40% 60% 80% 100%
connection strength

Figure S9. Time and dose-dependency of profiles. (A) Drug-drug matrix of the absolute difference in connection strengths as determined by MorphEUS analysis between low and high dose profiles with JSF-2019 applied. (B) Broad categorization of bedaquiline, moxifloxacin, rifampicin, and isoniazid at 6 hours mapped onto the joint dose profiles at ~1 doubling time (Fig. 3). The 17-hour profiles are shown as a point of comparison.

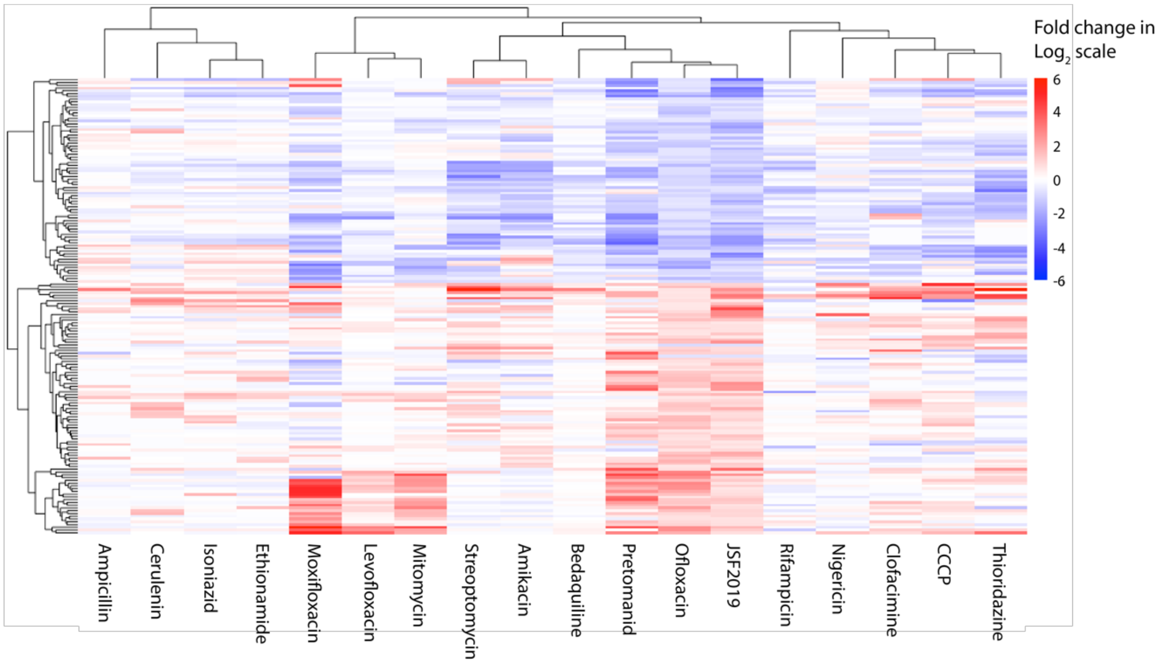

Fig. S10. Transcriptional analysis of 165 genes co-regulated by JSF-2019 and ofloxacin. Hierarchical clustering by Pearson distance of the gene expression profiles for 165 genes that were significantly co-regulated by JSF-2019 and ofloxacin by >1.5-fold.

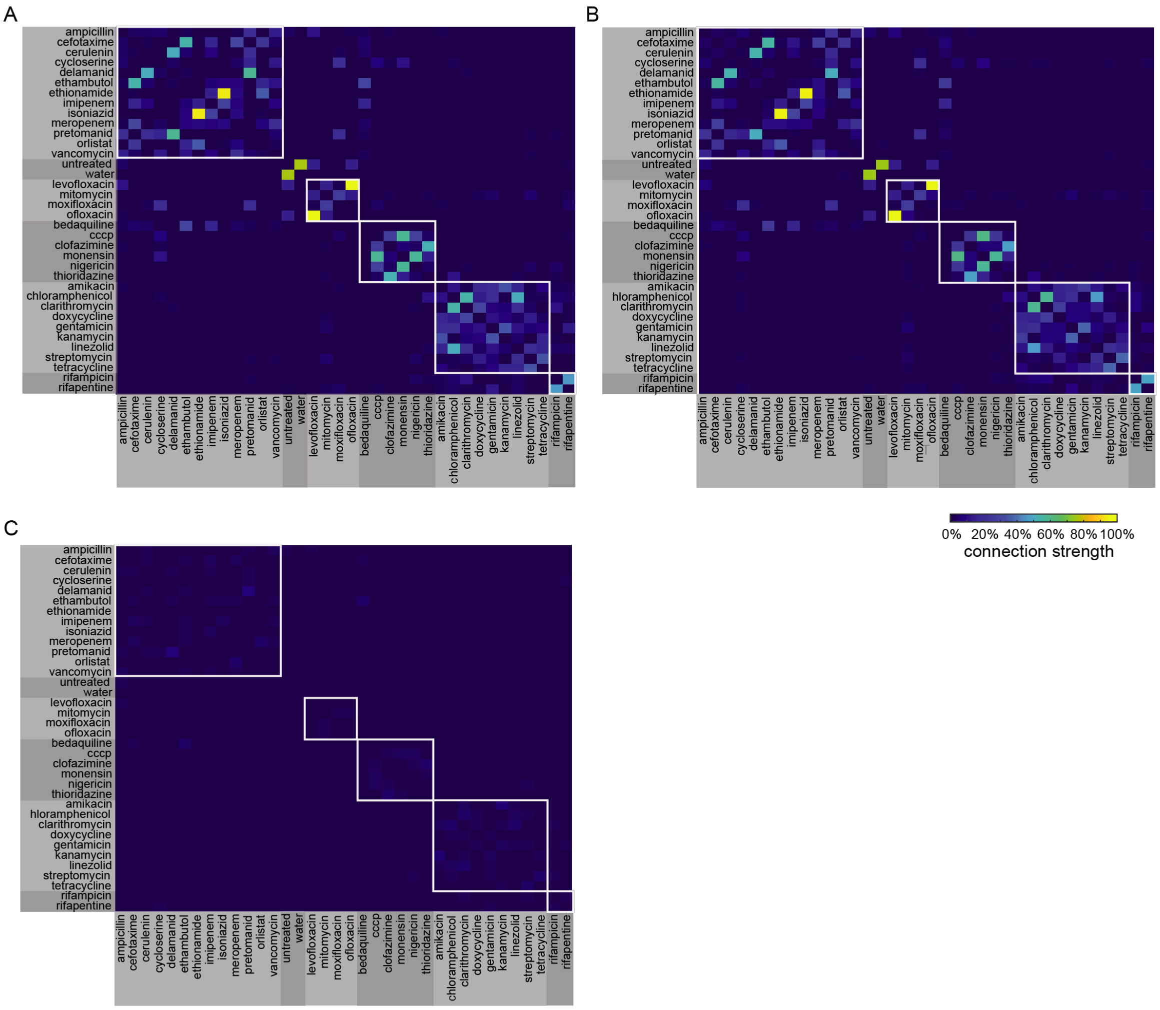

Fig. S11. 70 classification trials are sufficient for cKNN convergence. cKNN matrices with 70 (A, also shown in Fig. 3B) and 140 (B) classification trials. The magnitude of the difference in connection strengths are shown in (C).

Table S1. Compounds used in this study. Primary drug targets, off-target effects, and broad categorizations based on published studies. We determined IC90 values as the minimal concentration of drug needed to inhibit at least 90% of growth. The broad categorization of each drug was assigned based on literature review.

Drug name	MIC90 (µg/ml)	Solvent	Vendor	Catalog number	Primary target	Off target effects	Reference
**CELL WALL SYNTHESIS**							
Meropenem	25	DMSO	SigmaAldrich	1392454	Peptidoglycan (beta lactam)	ATP burst	(4, 5)
Ampicillin	25	DMSO	SigmaAldrich	A9393	Peptidoglycan (beta lactam)		(6)
Cefotaxime	25	Water	SigmaAldrich	C7039	Peptidoglycan (beta lactam)		(7)
Isoniazid	0.047	DMSO	SigmaAldrich	I3377	FASII/mycolic acid synthesis (InhA)	ATP burst	(5, 8)
Ethambutol	1.5	DMSO	Alfa Aesar	J60695	Arabinogalactan (AftB,AftC,AftD, EmbC)	ATP burst	(5, 8)
Ethionamide	3.125	DMSO	TCI Chemicals	E0695	FASII/mycolic acid synthesis (InhA)		(8)
Imipenem	3.125	Water	SigmaAldrich	PHR1796	Peptidoglycan (beta lactam)	ATP burst	(7, 9)
Vancomycin	25	Water	SigmaAldrich	V0045000	Peptidoglycan (d-ala-d crosslinking)		(8)
Cycloserine	6.25	DMSO	Calbiochem	239831	Peptidoglycan (d-ala-d ligase)		(8, 10)
Delamanid	1.5	DMSO	Advanced ChemBlocks Inc	L13485	Respiratory toxicity/mycobacterial cell wall (Nitroimidazole)		(11-13)
Pretomanid	12.5	DMSO	ApexBio Technology	A1736	Respiratory toxicity/mycobacterial cell wall (Nitroimidazole)	NO release	(11, 13, 14)

Cerulenin	12.5	DMSO	SigmaAldrich	C2389	FASII (KasA, KasB)		(8)
Orlistat	50 μM*	DMSO	VWR	89149-186	PDIM		(15)
**DNA SYNTHESIS**							
Levofloxacin	3.125	DMSO	SigmaAldrich	28266	DNA gyrase		(16, 17)
Moxifloxacin	6.25	DMSO	Alfa Aesa	J66626	DNA gyrase		(18)
Mitomycin	12.5	DMSO	SigmaAldrich	Y0000378	Alkylation of DNA	Redox recycling	(19, 20)
Ofloxacin	12.5	1N NaOH	SigmaAldrich	O8757	DNA gyrase		(16, 17, 21)
**PROTEIN SYNTHESIS**							
Kanamycin	12.5	Water	VWR	408	Aminoglycoside; 30S ribosomal subunit		(22, 23)
Amikacin	6.25	Water	SigmaAldrich	A3650	Aminoglycoside; 30S ribosomal subunit		(22, 23)
Chloramphenicol	25	DMSO	SigmaAldrich	C0378	50S ribosomal subunit		(24)
Clarithromycin	12.5	DMSO	SigmaAldrich	A3487	50S ribosomal subunit		(23)
Doxycycline	12.5	DMSO	SigmaAldrich	D9891	30S ribosomal subunit		(25, 26)
Gentamycin	12.5	Water	SigmaAldrich	G1264	Aminoglycoside; 30S ribosomal subunit		(22, 27)
Streptomycin	3.125	Water	SigmaAldrich	S6501	Aminoglycoside; 30S ribosomal subunit		(28, 29)
Tetracycline	50	DMSO	SigmaAldrich	87128	30S ribosomal subunit		(25)
Linezolid	3.125	DMSO	ApexBio Technology	A5181	50S ribosomal subunit		(30)
**RESPIRATION**							
Carbonyl cyanide 3-chlorophenyl-hydrazone	6.25	DMSO	SigmaAldrich	C2759	Proton motive force		(31)
Monensin	12.5	MeOH	SigmaAldrich	M5273	Proton motive force		(32)
Nigericin	6.25	MeOH	SigmaAldrich	N7143	Proton motive force		(33)

Thioridazine	25	DMSO	Enzo Life Sciences	BML-NS835-0005	Electron transport chain - NADH dehydrogenase (NDH-2)		(33-35)
Pyrazinamide	50*	DMSO	TCI Chemicals	P0633	Proton motive force, fatty acid synthesis, trans-translation, coenzyme A synthesis		(34, 36, 37)
Clofazimine	6.25	DMSO	SigmaAldrich	C8895	Electron transport chain - NDH-2		(34, 38)
Bedaquiline	3.125	DMSO	SigmaAldrich	10288-25MG	ATP synthase		(5, 34, 39-41)
**RNAP**							
Rifapentine	0.09	DMSO	ApexBio Technology	B2127	RpoB		(42)
Rifampicin	0.09	DMSO	TCI Chemicals	R0079	RpoB		(43)
**Blinded Compounds**							
DG167	0.39 μM	DMSO	N/A	N/A	KasA		(44)
JSF-3285	0.2 μM	DMSO	N/A	N/A	KasA		(45)
JSF-2019	0.15 μM	DMSO	N/A	N/A	InhA (FAS-II)		(46)

*Drugs that did not reach IC90

Table S2. Features included in analysis pipeline. For each feature, with exception to FEATURE_1_count and FEATURE_2_count (noted in green), the median, quartile 1 (25%), quartile 3 (75%), and interquartile range were included as metrics, resulting in 94 total features. Descriptions come from the ImageJ documentation (47).

**Feature**	**Description**	**Unit**
**FEATURE_1_count**	number of times given cell fluoresced with SYTO 24 stain	number of instances
**FEATURE_2_count**	number of FM4-64FX stain positive foci	number of instances
**SHAPE_area**	area of cell	μm^2
**SHAPE_aspectRatio**	major axis/minor axis of cell	unitless
**SHAPE_circularity**	4*[area][perimeter]2 of cell<br>ranges from 0 (infinitely elongated polygon) to 1 (perfect circle)	unitless
**SHAPE_length**	length of cell	μm
**SHAPE_perimeter**	perimeter of cell	μm
**SHAPE_solidity**	[area][convex area] of cell	unitless
**SHAPE_width**	width of cell	μm
**f_INTENSITY**	intensity of FM4-64FX stain	relative fluorescence units (RFU)
**f_SHAPE_area**	area of FM4-64FX foci	μm^2
**f_SHAPE_aspectRatio**	major axis/minor axis of FM4-64FX foci	unitless
**f_SHAPE_circularity**	4*[area][perimeter]2 of FM4-64FX foci<br>ranges from 0 (infinitely elongated polygon) to 1 (perfect circle)	unitless
**f_SHAPE_length**	length of FM4-64FX foci	μm
**f_SHAPE_perimeter**	perimeter of FM4-64FX foci	μm
**f_SHAPE_solidity**	[area][convex area] of FM4-64FX foci	unitless

**f_SHAPE_width**	width of FM4-64FX foci	μm
**s_INTENSITY**	Intensity of SYTO 24 stain	relative fluorescence units (RFU)
**s_SHAPE_area**	area of SYTO 24 foci	μm^2
**s_SHAPE_aspectRatio**	major axis/minor axis of SYTO 24 foci	unitless
**s_SHAPE_circularity**	4*[area][perimeter]2 of SYTO 24 foci Ranges from 0 (infinitely elongated polygon) to 1 (perfect circle)	unitless
**s_SHAPE_length**	length of SYTO 24 foci	μm
**s_SHAPE_perimeter**	perimeter of SYTO 24 foci	μm
**s_SHAPE_solidity**	[area][convex area] of SYTO 24 foci	unitless
**s_SHAPE_width**	width of SYTO 24 foci	μm

Movie S1. Time-lapse imaging of *M. smegmatis* before, during, and after treatment with ethambutol. RpoB-EGFP reporter *M. smegmatis* was imaged in nutrient-rich growth conditions in a constant-flow microfluidic device for 10 hours with no antibiotics, followed by a 6-hour drug treatment with 9.375μg/ml of ethambutol, and followed by a 10-hour no drug recovery period.

Movie S2. Time-lapse imaging of *M. smegmatis* before, during, and after treatment with rifampicin. RpoB-EGFP reporter *M. smegmatis* was imaged in nutrient-rich growth conditions in a constant-flow microfluidic device for 10 hours with no antibiotics, following by a 6-hour drug treatment with 75μg/ml of rifampicin, and followed by a 10-hour no drug recovery period.

Movie S3. Time-lapse imaging of *M. smegmatis* before, during, and after treatment with moxifloxacin. RpoB-EGFP reporter *M. smegmatis* was imaged in nutrient-rich growth conditions in a constant-flow microfluidic device for 10 hours with no antibiotics, following by a 6-hour drug treatment with 0.781μg/ml of moxifloxacin, and followed by a 10-hour no drug recovery period.

Dataset S1 : Matrix of drug-drug connection strengths. MorphEUS output for the joint dose (sheet 1), low dose (sheet 2), and high dose drug profiles (sheet 3). Numerical values here correspond with the heatmap representations in Figure 3B and Figure S5B, respectively.

Dataset S2: List of co-regulated genes between JSF-2019 and ofloxacin. Table of genes used for hierarchical clustering in Figure S10. Functional annotation of each gene product was designated using tuberculist (http://tuberculist.epfl.ch).

References

1. A. U. Muller, F. Imkamp, E. Weber-Ban, The Mycobacterial LexA/RecA-Independent DNA Damage Response Is Controlled by PafBC and the Pup-Proteasome System. *Cell Rep* **23**, 3551-3564 (2018).
2. E. O. Davis, E. M. Dullaghan, L. Rand, Definition of the mycobacterial SOS box and use to identify LexA-regulated genes in Mycobacterium tuberculosis. *J Bacteriol* **184**, 3287-3295 (2002).
3. K. L. Smollett *et al.*, Global analysis of the regulon of the transcriptional repressor LexA, a key component of SOS response in Mycobacterium tuberculosis. *J Biol Chem* **287**, 22004-22014 (2012).
4. P. Kumar *et al.*, Meropenem inhibits D,D-carboxypeptidase activity in Mycobacterium tuberculosis. *Mol Microbiol* **86**, 367-381 (2012).
5. A. Shetty, T. Dick, Mycobacterial Cell Wall Synthesis Inhibitors Cause Lethal ATP Burst. *Front Microbiol* **9**, 1898 (2018).
6. H. F. Chambers *et al.*, Can penicillins and other beta-lactam antibiotics be used to treat tuberculosis? *Antimicrob Agents Chemother* **39**, 2620-2624 (1995).
7. V. Dubee *et al.*, Inactivation of Mycobacterium tuberculosis l,d-transpeptidase LdtMt(1) by carbapenems and cephalosporins. *Antimicrob Agents Chemother* **56**, 4189-4195 (2012).
8. K. A. Abrahams, G. S. Besra, Mycobacterial cell wall biosynthesis: a multifaceted antibiotic target. *Parasitology* **145**, 116-133 (2018).
9. M. Lindman, T. Dick, Bedaquiline Eliminates Bactericidal Activity of beta-Lactams against Mycobacterium abscessus. *Antimicrob Agents Chemother* **63** (2019).
10. J. B. Bruning, A. C. Murillo, O. Chacon, R. G. Barletta, J. C. Sacchettini, Structure of the Mycobacterium tuberculosis D-alanine:D-alanine ligase, a target of the antituberculosis drug D-cycloserine. *Antimicrob Agents Chemother* **55**, 291-301 (2011).
11. S. Wellington, D. T. Hung, The Expanding Diversity of Mycobacterium tuberculosis Drug Targets. *ACS Infect Dis* **4**, 696-714 (2018).

12. M. Matsumoto *et al.*, OPC-67683, a nitro-dihydro-imidazooxazole derivative with promising action against tuberculosis in vitro and in mice. *PLoS Med* **3**, e466 (2006).

13. U. Manjunatha, H. I. Boshoff, C. E. Barry, The mechanism of action of PA-824: Novel insights from transcriptional profiling. *Commun Integr Biol* **2**, 215-218 (2009).

14. C. K. Stover *et al.*, A small-molecule nitroimidazopyran drug candidate for the treatment of tuberculosis. *Nature* **405**, 962-966 (2000).

15. C. Rens *et al.*, Effects of Lipid-Lowering Drugs on Vancomycin Susceptibility of Mycobacteria. *Antimicrob Agents Chemother* **60**, 6193-6199 (2016).

16. S. Akcali, S. Surucuoglu, C. Cicek, B. Ozbakkaloglu, In vitro activity of ciprofloxacin, ofloxacin and levofloxacin against Mycobacterium tuberculosis. *Ann Saudi Med* **25**, 409-412 (2005).

17. N. Rastogi, K. S. Goh, A. Bryskier, A. Devallois, In vitro activities of levofloxacin used alone and in combination with first- and second-line antituberculous drugs against Mycobacterium tuberculosis. *Antimicrob Agents Chemother* **40**, 1610-1616 (1996).

18. K. J. Aldred, T. R. Blower, R. J. Kerns, J. M. Berger, N. Osheroff, Fluoroquinolone interactions with Mycobacterium tuberculosis gyrase: Enhancing drug activity against wild-type and resistant gyrase. *Proc Natl Acad Sci U S A* **113**, E839-846 (2016).

19. Y. Wang *et al.*, Distinct roles of cytochrome P450 reductase in mitomycin C redox cycling and cytotoxicity. *Mol Cancer Ther* **9**, 1852-1863 (2010).

20. K. Lin *et al.*, Mycobacterium tuberculosis Thioredoxin Reductase Is Essential for Thiol Redox Homeostasis but Plays a Minor Role in Antioxidant Defense. *PLoS Pathog* **12**, e1005675 (2016).

21. M. Imamura, S. Shibamura, I. Hayakawa, Y. Osada, Inhibition of DNA gyrase by optically active ofloxacin. *Antimicrob Agents Chemother* **31**, 325-327 (1987).

22. L. P. Kotra, J. Haddad, S. Mobashery, Aminoglycosides: perspectives on mechanisms of action and resistance and strategies to counter resistance. *Antimicrob Agents Chemother* **44**, 3249-3256 (2000).

23. W. W. Barrow, Treatment of mycobacterial infections. *Rev Sci Tech* **20**, 55-70 (2001).

24. J. M. Schifano *et al.*, Mycobacterial toxin MazF-mt6 inhibits translation through cleavage of 23S rRNA at the ribosomal A site. *Proc Natl Acad Sci U S A* **110**, 8501-8506 (2013).

25. I. Chopra, M. Roberts, Tetracycline antibiotics: mode of action, applications, molecular biology, and epidemiology of bacterial resistance. *Microbiol Mol Biol Rev* **65**, 232-260 ; second page, table of contents (2001).

26. N. Alsaad *et al.*, Potential antimicrobial agents for the treatment of multidrug-resistant tuberculosis. *Eur Respir J* **43**, 884-897 (2014).

27. F. Sanz-Garcia *et al.*, Mycobacterial Aminoglycoside Acetyltransferases: A Little of Drug Resistance, and a Lot of Other Roles. *Front Microbiol* **10**, 46 (2019).

28. R. Chulluncuy, C. Espiche, J. A. Nakamoto, A. Fabbretti, P. Milon, Conformational Response of 30S-bound IF3 to A-Site Binders Streptomycin and Kanamycin. *Antibiotics (Basel)* **5** (2016).

29. M. S. Shaila, K. P. Gopinathan, T. Ramakrishnan, Protein synthesis in Mycobacterium tuberculosis H37Rv and the effect of streptomycin in streptomycin-susceptible and -resistant strains. *Antimicrob Agents Chemother* **4**, 205-213 (1973).

30. D. M. Livermore, Linezolid in vitro: mechanism and antibacterial spectrum. *J Antimicrob Chemother* **51 Suppl 2**, ii9-16 (2003).

31. C. M. Pule *et al.*, Efflux pump inhibitors: targeting mycobacterial efflux systems to enhance TB therapy. *J Antimicrob Chemother* **71**, 17-26 (2016).

32. A. Huczynski, J. Janczak, D. Lowicki, B. Brzezinski, Monensin A acid complexes as a model of electrogenic transport of sodium cation. *Biochim Biophys Acta* **1818**, 2108-2119 (2012).

33. P. A. Black *et al.*, Energy metabolism and drug efflux in Mycobacterium tuberculosis. *Antimicrob Agents Chemother* **58**, 2491-2503 (2014).
34. D. Bald, C. Villellas, P. Lu, A. Koul, Targeting Energy Metabolism in Mycobacterium tuberculosis, a New Paradigm in Antimycobacterial Drug Discovery. *MBio* **8** (2017).
35. H. I. Boshoff *et al.*, The transcriptional responses of Mycobacterium tuberculosis to inhibitors of metabolism: novel insights into drug mechanisms of action. *J Biol Chem* **279**, 40174-40184 (2004).
36. E. A. Lamont, N. A. Dillon, A. D. Baughn, The Bewildering Antitubercular Action of Pyrazinamide. *Microbiol Mol Biol Rev* **84** (2020).
37. Y. Zhang, M. M. Wade, A. Scorpio, H. Zhang, Z. Sun, Mode of action of pyrazinamide: disruption of Mycobacterium tuberculosis membrane transport and energetics by pyrazinoic acid. *J Antimicrob Chemother* **52**, 790-795 (2003).
38. T. Yano *et al.*, Reduction of clofazimine by mycobacterial type 2 NADH:quinone oxidoreductase: a pathway for the generation of bactericidal levels of reactive oxygen species. *J Biol Chem* **286**, 10276-10287 (2011).
39. G. G. Jickky Palmae Sarathy, Thomas Dick, Re-Understanding the Mechanisms of Action of the Anti-Mycobacterial Drug Bedaquiline. *Antibiotics* **8** (2019).
40. A. Koul *et al.*, Delayed bactericidal response of Mycobacterium tuberculosis to bedaquiline involves remodelling of bacterial metabolism. *Nat Commun* **5**, 3369 (2014).
41. B. S. Lee *et al.*, Inhibitors of energy metabolism interfere with antibiotic-induced death in mycobacteria. *J Biol Chem* **294**, 1936-1943 (2019).
42. S. S. Munsiff, C. Kambili, S. D. Ahuja, Rifapentine for the treatment of pulmonary tuberculosis. *Clin Infect Dis* **43**, 1468-1475 (2006).
43. K. Richardson *et al.*, Temporal and intrinsic factors of rifampicin tolerance in mycobacteria. *Proc Natl Acad Sci U S A* **113**, 8302-8307 (2016).

44. P. Kumar *et al.*, Synergistic Lethality of a Binary Inhibitor of Mycobacterium tuberculosis KasA. *MBio* **9** (2018).

45. D. Inoyama *et al.*, A Preclinical Candidate Targeting Mycobacterium tuberculosis KasA. *Cell Chem Biol* 10.1016/j.chembiol.2020.02.007 (2020).

46. X. Wang *et al.*, Antitubercular Triazines: Optimization and Intrabacterial Metabolism. *Cell Chem Biol* 10.1016/j.chembiol.2019.10.010 (2019).

47. T. Peng *et al.*, A BaSiC tool for background and shading correction of optical microscopy images. *Nat Commun* **8**, 14836 (2017).
